# Pangenomics Analysis Reveals Diversification of Enzyme Families and Niche Specialization in Globally Abundant SAR202 Bacteria

**DOI:** 10.1128/mBio.02975-19

**Published:** 2020-01-07

**Authors:** Jimmy H. W. Saw, Takuro Nunoura, Miho Hirai, Yoshihiro Takaki, Rachel Parsons, Michelle Michelsen, Krista Longnecker, Elizabeth B. Kujawinski, Ramunas Stepanauskas, Zachary Landry, Craig A. Carlson, Stephen J. Giovannoni

**Affiliations:** a Oregon State University, Corvallis, Oregon, USA; b Department of Biological Sciences, George Washington University, Washington, DC, USA; c Research Center for Bioscience and Nanoscience (CeBN), Japan Agency for Marine-Earth Science and Technology (JAMSTEC), Yokosuka, Kanagawa, Japan; d Super-cutting-edge Grand and Advanced Research (SUGAR) Program, Institute for Extra-cutting-edge Science and Technology Avant-garde Research (X-star), Japan Agency for Marine-Earth Science and Technology (JAMSTEC), Yokosuka, Kanagawa, Japan; e Bermuda Institute for Ocean Science (BIOS), St. Georges, Bermuda; f Woods Hole Oceanographic Institution, Woods Hole, Massachusetts, USA; g Bigelow Laboratory for Ocean Sciences, East Boothbay, Maine, USA; h ETH Zürich, Zurich, Switzerland; i Marine Science Institute, University of California Santa Barbara, Santa Barbara, California, USA; j Department of Ecology, Evolution, and Marine Biology, University of California Santa Barbara, Santa Barbara, California, USA; University of Oklahoma

**Keywords:** SAR202, biological carbon pump, carbon sequestration, dissolved organic matter, enolase, marine carbon cycle, recalcitrant organic matter

## Abstract

It has been hypothesized that the abundant heterotrophic ocean bacterioplankton in the SAR202 clade of the phylum Chloroflexi evolved specialized metabolisms for the oxidation of organic compounds that are resistant to microbial degradation via common metabolic pathways. Expansions of paralogous enzymes were reported and implicated in hypothetical metabolism involving monooxygenase and dioxygenase enzymes. In the proposed metabolic schemes, the paralogs serve the purpose of diversifying the range of organic molecules that cells can utilize. To further explore SAR202 evolution and metabolism, we reconstructed single amplified genomes and metagenome-assembled genomes from locations around the world that included the deepest ocean trenches. In an analysis of 122 SAR202 genomes that included seven subclades spanning SAR202 diversity, we observed additional evidence of paralog expansions that correlated with evolutionary history, as well as further evidence of metabolic specialization. Consistent with previous reports, families of flavin-dependent monooxygenases were observed mainly in the group III SAR202 genomes, and expansions of dioxygenase enzymes were prevalent in those of group VII. We found that group I SAR202 genomes encode expansions of racemases in the enolase superfamily, which we propose evolved for the degradation of compounds that resist biological oxidation because of chiral complexity. Supporting the conclusion that the paralog expansions indicate metabolic specialization, fragment recruitment and fluorescent *in situ* hybridization (FISH) with phylogenetic probes showed that SAR202 subclades are indigenous to different ocean depths and geographical regions. Surprisingly, some of the subclades were abundant in surface waters and contained rhodopsin genes, altering our understanding of the ecological role of SAR202 species in stratified water columns.

## Abstract (precis)

The oceans contain an estimated 662 Pg C in the form of dissolved organic matter (DOM). Information about microbial interactions with this vast resource is limited, despite broad recognition that DOM turnover has a major impact on the global carbon cycle. To explain patterns in the genomes of marine bacteria, we propose hypothetical metabolic pathways for the oxidation of organic molecules that are resistant to oxidation via common pathways. The hypothetical schemes we propose suggest new metabolic pathways and classes of compounds that could be important for understanding the distribution of organic carbon throughout the biosphere. These genome-based schemes will remain hypothetical until evidence from experimental cell biology can be gathered to test them. Our findings also fundamentally change our understanding of the ecology of SAR202 bacteria, showing that metabolically diverse variants of these cells occupy niches spanning all depths and are not relegated to the dark ocean.

## INTRODUCTION

SAR202 species comprise the most abundant lineage of bacteria in the deep oceans. This clade diversified approximately 2 billion years ago, forming seven subclades referred to as groups I to VII (1, 2). The first report on SAR202 used molecular data to demonstrate that their relative abundance increased dramatically at the transition between the euphotic and aphotic zones of the oceans (3). An early study that applied rRNA fluorescent *in situ* hybridization (FISH) probes for the SAR202 clade showed that they constitute, on average, about 10% of total bacterioplankton throughout the mesopelagic of the Sargasso Sea, Central Pacific Ocean, and Eastern Pacific coastal waters (4). A later study that also applied FISH found that SAR202 species comprise up to 5% of the total bacterioplankton community in the epipelagic zone and up to 30% in the mesopelagic and bathypelagic zones in realms of the Atlantic Ocean (5).

Microbes adapted to dark ocean regions (mesopelagic, 200 to 1,000 m; bathypelagic, 1,000 to 4,000 m; abyssopelagic, 4,000 to 6,000 m; hadalpelagic, 6,000 to 11,000 m) exploit environments where the most abundant energy resources are sinking organic particles or recalcitrant dissolved organic matter (DOM). These recalcitrant compounds are mainly remnants from primary production in the epipelagic zone, which are attenuated in transit through food webs. In the dark oceans, low-level primary production also occurs locally, fueled by chemolithoautotrophy (6). As labile DOM and recalcitrant DOM are reprocessed by microbes, a fraction is chemically altered to forms that resist or escape microbial degradation, leading to the accumulation of refractory dissolved organic matter (RDOM), which has a residence time of thousands of years (7). These ideas are captured in the microbial carbon pump (MCP) conceptual model (8).

SAR202 species have escaped cultivation to date. Insight into their metabolism has come from field studies and comparative genomics (9). Recent studies, using both single-cell and metagenomic sequencing, have highlighted the differing roles for SAR202 groups at sites around the world. One study assembled three nearly complete SAR202 metagenome-assembled genomes (MAGs) from metagenomes from oxygen minimum zones in the Gulf of Mexico and observed expression of nitrate reductase genes, suggesting these cells have the capacity for anaerobic respiration (10). Another study investigated vertical stratification and found that several SAR202 genomes encode genes for utilization of organosulfur compounds (11). An investigation of SAR202 from the Arctic Ocean described expanded families of dioxygenase enzymes that were proposed to function in aromatic compound degradation, potentially utilizing organic matter discharged from terrestrial sources (12). Freshwater relatives of SAR202 have also been discovered, shedding light on the diversity and ecology of *Chloroflexi* in aquatic habitats (13).

In a recent study of group III SAR202 species, we identified expansions of paralogous protein families, including powerful oxidative enzymes that we hypothesize could play a role in degrading recalcitrant DOM (2). SAR202 flavin-dependent monooxygenases (FMNOs) were proposed to oxidize a variety of chemically stable organic molecules by introducing single oxygen atoms, e.g., by oxidizing sterols and hopanoids to carboxyl-rich alicyclic molecules (CRAM) (2). CRAMs, which consist of fused aromatic and heterocyclic rings decorated with carboxyl groups, have been identified as an abundant class of RDOM molecules (14–16).

In this study, we investigated paralogous gene expansions and gene co-occurrence in a larger sample of SAR202 diversity. We reconstructed 10 new single amplified genomes (SAGs) from mesopelagic and hadal waters from the Northwestern Pacific Ocean, 11 new MAGs from the Bermuda Atlantic Time-series Study (BATS) site in the Sargasso Sea, and 62 new MAGs from *Tara* Oceans expedition metagenomes, for a total of 83 new SAR202 genomes. We also investigated the biogeography of these genomes and their distributions as a function of depth in the water column. Interpreting this information, we hypothesize that SAR202 evolved and diversified into multiple niches that involve the oxidation of recalcitrant compounds.

## RESULTS

### Overview of genomic bins and SAGs.

The total number of SAGs and MAGs in this study was 122, of which 83 are new and the remainder are from previous studies (2, 10, 17–19). Ten new SAR202 SAGs were obtained from three deep ocean trench stations in the Mariana, Ogasawara, and Japan trenches. Sixty-two new SAR202 MAGs were reconstructed from *Tara* Oceans metagenome reassemblies in this study. *Tara* metagenomic samples from different depths were assembled separately to help us preserve depth information for each MAG. Eleven new SAR202 MAGs came from metagenomic samples obtained at the Bermuda Atlantic Time-series Study (BATS) site. A table summarizing the origins and depths of samples from which the SAGs and MAGs were obtained is provided as Data Set S1.

10.1128/mBio.02975-19.10 DATA SET S1 Supplemental Excel file containing the following items: (i) SAG and MAG genome size, completeness, contamination, G+C content, accession numbers of genomes, environmental metadata, and enzyme abundance counts for certain overrepresented enzymes, (ii) metadata and accession numbers of *Tara* metagenomes used for assembly, binning, and read mapping, (iii) metadata and accession numbers of BATS metagenomes used for assembly, binning, and read mapping, (iv) accession numbers of deep ocean trench metagenomes used for read mapping, (v) 50 most abundant clusters of orthologous groups (COG) functional categories, (vi) fluorescent in situ hybridization (FISH) probes used to detect SAR202 groups I, II, and III, (vii) FISH dissociation data. Download Data Set S1, XLSX file, 0.04 MB. Copyright © 2019 Saw et al. 2019 Saw et al. This content is distributed under the terms of the Creative Commons Attribution 4.0 International license.

### SAR202 diversity revealed through phylogenomic analyses.

**FIG 1:**
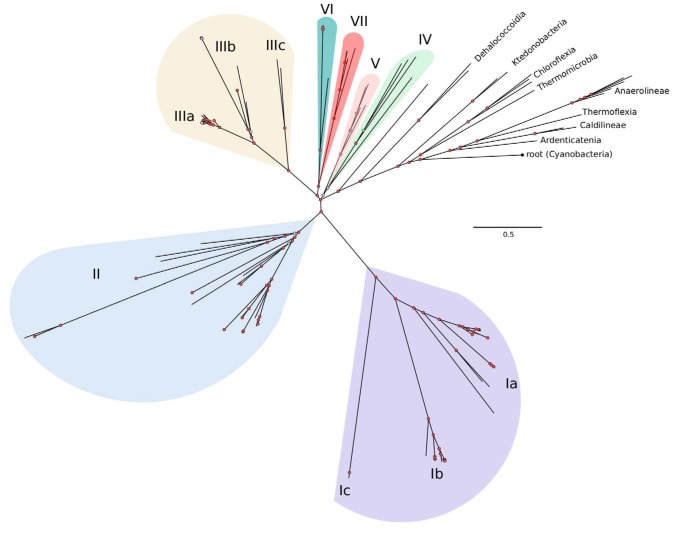
Phylogenomic tree of SAR202 genomes, built using 36 concatenated single-copy ChloNOGs. Phylogenomic inference was performed using PhyloBayes MPI version 1.7. Cyanobacterial sequences were used as the outgroup. Color shading identifies SAR202 groups used in subsequent figures. Detailed tree showing all tip labels are available on figshare (https://doi.org/10.6084/m9.figshare.8478227).

A phylogenomic tree was constructed from 36 concatenated single-copy genes that were selected based on their broad presence in genomes, suggesting core functions, and evidence of linear inheritance (Fig. 1). Using the ChloNOG (*Chloroflexi* nonsupervised orthologous groups) subset of gene clusters from the eggNOG database, we identified 639 orthologous gene clusters that are present as single copies in 141 genomes (122 SAR202, 17 other *Chloroflexi*, and 2 cyanobacteria genomes used as an outgroup).

The phylogenomic tree supported earlier findings showing that SAR202 members comprise a deeply branching monophyletic group that radiates from within the *Chloroflexi* and is possibly sister to the Dehalococcoides (Fig. 1). Several deeply branching subclades, groups IV to VII, radiate near the base of the clade. Groups III, II, and I appear in that order, ascending from the root. They are separated by large evolutionary distances and are the most abundantly represented SAR202 subgroups (Data Set S1). Detailed proposals to assign names to the major clades identified within the SAR202 radiation can be found in the supplemental material.

### Overview of paralogous enzyme superfamilies in SAR202.

**FIG 2:**
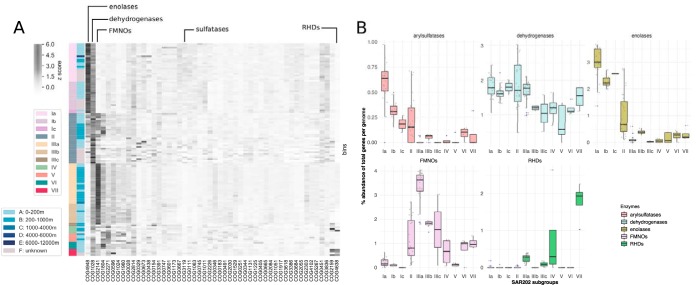
(A) Heatmap of the most abundant COG categories in SAR202 genomes categorized by subgroups. The first column of color bars indicates different SAR202 subgroups, and the second column of color bars indicate the depth of samples from which the SAGs or MAGs were obtained. The grayscale gradient indicates Z scores of percent abundance of total number of genes. (B) Distribution of the major paralog expansions among the SAR202 subgroups.

Paralog expansions, especially diverse, ancient ones, can indicate past evolutionary events in which new enzyme activities were vehicles for niche expansions. To investigate paralog expansions across SAR202 genomes, we constructed a heatmap showing relative abundances of the 50 most abundant clusters of orthologous groups (COG) categories (Fig. 2A). The heatmap revealed five major expansions of paralogous gene families and many other less prominent expansions. The distributions of these groups of paralogs across the major SAR202 subclades are shown in Fig. 2B. COG4948 proteins, the enolase superfamily, were mainly found in groups I and II (Fig. 2B); COG2141 proteins, the SAR202 FMNO paralogs, were found mainly in groups II and III, but some paralogs were also detected in groups IV, VI, and VII; and COG4638, the ring-hydroxylating dioxygenase paralogs, were found mostly in group VII. Ring-hydroxylating dioxygenases were also previously identified in SAR202 species, but they were found in a different SAR202 member unrelated to the group VII species described in this study (12).

**FIG 3:**
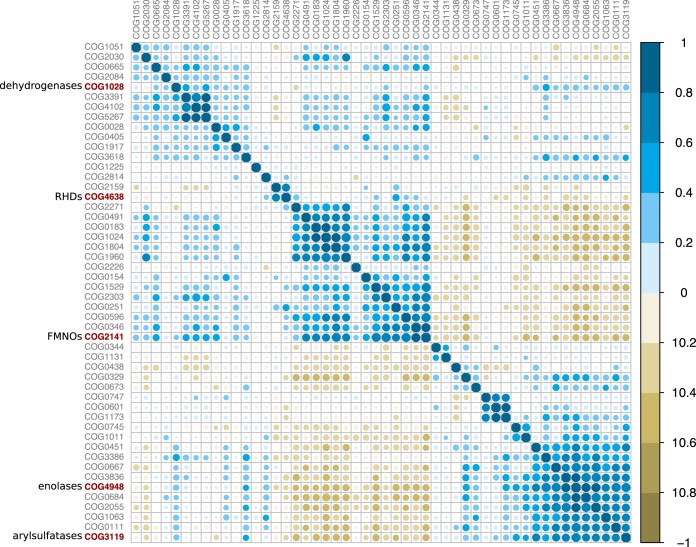
Correlations among top 50 most abundant COG functional categories, demonstrating that the major paralog expansions identified in Fig. 2 are linked to other expanded families of proteins, indicating metabolic specialization.

A correlation matrix of the 50 most abundant COG categories showed that the expansions of the five major paralog families discussed above are linked to broad shifts in metabolism (Fig. 3). For example, COG3391, COG4102, and COG5267 all represent uncharacterized conserved proteins. COG0747, COG0601, and COG1173 represent components involved in dipeptide transport. We interpret these patterns as evidence that the ancient paralog expansions described above accompanied metabolic reorganization and specialization in the SAR202 subclades.

### The diversification of flavin-dependent monooxygenases in group III.

An expansion and radiation of diverse FMNO members in group III SAR202 species was previously reported (2). We found further support for this conclusion in this broader analysis of SAR202 diversity and also observed elevated numbers of FMNO paralogs in groups II, IV, VI. and VII (Fig. 2). The number of paralogous FMNOs can be >100 in some genomes (the largest number identified was 114 copies in a single genome), with members of group IIIa encoding the largest numbers and the greatest relative abundances of FMNOs, up to 4% when normalized to total number of genes per genome (Fig. 2B). FMNOs were also present in other SAR202 subgroups at lower copy numbers. Group I members encode the fewest copies of FMNOs; in some genomes this number approaches zero. The five most abundant FMNOs were annotated as alkanal mono-oxygenase alpha chain (23% of all annotations), limonene 1,2-monooxygenase (21%); phthiodiolone/phenolphthiodiolone dimycocerosates ketoreductase (13.9%), F420-dependent glucose-6-phosphate dehydrogenase (13.7%), and alkanesulfonate monooxygenase (7.2%).

**FIG 4:**
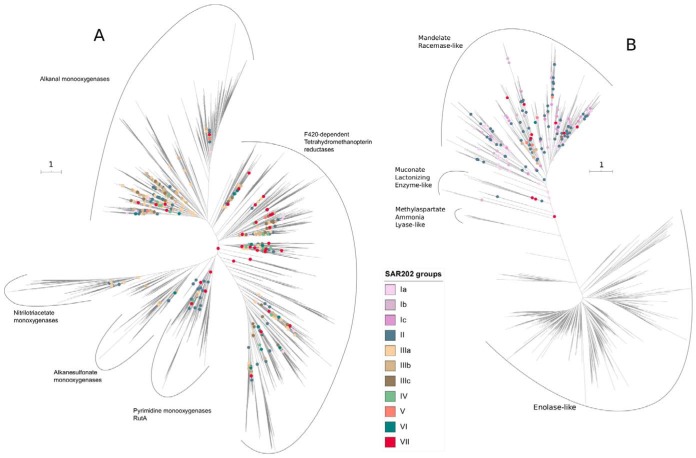
(A) Phylogenetic tree of the FMNO superfamily of enzymes. Internal nodes marked with colored circles indicate points of attachment for SAR202 lineages. The deep positions of the SAR202 nodes suggest that a substantial part of enzyme diversity in the FMNO superfamily is found in SAR202. The cluster of group IIIa nodes deep in the alkanal monooxygenase subclade suggest that these enzymes, in particular, may have evolved in SAR202. (B) Phylogenetic tree of the enolase superfamily of enzymes. SAR202 paralogs branch deeply and are confined to the madelate racemase-like enzyme subfamily of enolases. Scale bar represents the number of amino acid substitutions.

Because automatic annotation can sometimes fail to assign proper function to the genes, we built a maximum likelihood (ML) phylogenetic tree of all FMNOs identified in databases to better visualize the functional diversity of the FMNOs (Fig. 4A). We identified the following five broadly classified functional groups: F420-dependent tetrahydromethanopterin reductases, alkanal monooxygenases, nitrilotriacetate monooxygenases, alkanesulfonate monooxygenases, and pyrimidine monooxygenases (RutA). Most fall into the alkanal and F420-dependent monooxygenases. The SAR202 F420-dependent monooxygenases are highly diverse and appear to be paraphyletic.

Type II Baeyer-Villiger monooxygenases were found in group IIIa SAR202 species, as described previously (2), and fall into the broad category of alkanal monooxygenases. The alkanal monooxygenases formed a monophyletic clade with deepest nodes belonging to group IIIa genes (Fig. 4A). This pattern indicates that this subfamily of enzymes may have originated within SAR202 group IIIa.

### The group I and group II enolase paralog expansion may be an adaptation to unlock chiral diversity in DOM resources.

We observed an expansion of diverse enolase superfamily paralogs in groups I and II (Fig. 2A and B, and 4B). The presence of enolase paralogs in SAR202 genomes was first noted in MAGs obtained from a northern Gulf of Mexico “dead zone” (10). The annotations of the five most abundant SAR202 enolases are d-galactonate dehydratase (52.9% of all annotations), l-rhamnonate dehydratase (16.4%), starvation-sensing protein RspA (10%), mandelate racemase (6.8%), and l-Ala-d/l-Glu epimerase (5.4%).

The numbers of enolase paralogs in group I members ranged from 4 to 75 (1.3 to 3.5% of total genes found in each subclade); other SAR202 clades appear to encode very few copies of this enzyme (Fig. 2B), with the exception of group II SAR202, which encode both FMNO and enolase paralogs in roughly equal abundances (Fig. 2B). Enzymes of the enolase superfamily catalyze mechanistically diverse reactions such as racemizations, epimerizations, β-eliminations of hydroxyl or amino groups, and cycloisomerizations, but all known reactions catalyzed by these enzymes involve abstraction of an α-proton from carbons adjacent to carboxylic acid groups and stabilization of the enolate anion intermediate through a divalent metal ion, usually Mg^2+^ (20, 21).

Muconate cycloisomerases were also detected in SAR202 members, although they constitute a small fraction of the enolases found. They belong to the muconate lactonizing enzyme (MLE) family, which are involved in the conversion of lignin-derived aromatic compounds, catechols, and protocatechuate, to intermediates used in the citric acid cycle (22, 23). It is worth noting that, although members of groups I and II encode the greatest diversity of enolase family enzymes, some group III members also encode a few of these genes, the majority of which are mandelate racemases (Fig. 2B and 4B).

A phylogenetic tree was constructed to highlight the diversity and functions of enolase family enzymes found in group I SAR202 genomes. Enzymes within this superfamily can be divided into four categories, namely, enolases, mandelate racemases, muconate lactonizing enzymes, and methylaspartate ammonia lyases (Fig. 4B). Nearly all of the enolases in SAR202 belong to the mandelate racemase family. Enzymes within this family include mandelate racemase, galactonate dehydratase, glucarate dehydratase, idarate dehydratase, and similar enzymes that can either interconvert two stereoisomers or perform dehydration reactions (20).

Enzymes that interconvert *R* and *S* forms (stereoisomers) could potentially improve the fitness of heterotrophs by enabling them to access chiral complexity. For example, organisms that encode mandelate racemase (MR) in their genomes can interconvert between (*R*)-mandelate and (*S*)-mandelate, the latter of which is the first compound in the mandelate and hydroxy-mandelate degradation pathways (24). We postulate that the expansion of diverse enolase superfamily paralogs in groups I and II is an adaptation to metabolize organic compounds that are recalcitrant to oxidation because of chiral complexity. An alternative hypothesis is that these enzymes participate in the biosynthesis of compounds of mixed chirality. In the discussion section, we further explore the potential ramifications of these hypotheses.

### Sulfatases in group I and II members.

Sulfatases in SAR202 were first reported in a study on the oxygen-deficient dead zones in the Gulf of Mexico (10). We also detected a large number of genes belonging to COG3119 (arylsulfatase A [AslA]) and related enzymes classified in “inorganic ion transport and metabolism,” predominantly in group I and II members (Fig. 2B). Arylsulfatases and choline sulfatases can hydrolyze sulfated polysaccharides such as fucoidan, which is produced by marine eukaryotes (algae or fungi). These enzymes are expressed intracellularly by a species of marine fungus (25) and are also found in marine Rhodobacteraceae species that are mutualists of marine eukaryotes (26). Marine brown algae such as Macrocystis and Sargassum are known to produce fucoidans, which consist of α-l-fucosyl monomers (27). We speculate that SAR202 groups I and II could be utilizing arylsulfatases to break down similar sulfated polysaccharides produced by algae in the upper water column.

### Ring-hydroxylating dioxygenases in group VII: a molecular arsenal to break down aromatic compounds?

In group VII, we found an expanded family of genes related to COG4638, annotated as “phenylpropionate dioxygenases or related ring-hydroxylating dioxygenases, large terminal subunit.” Enzymes belonging to the ring-hydroxylating dioxygenase (RHD) family occur as monomers of subunits alpha and beta (α_2_β_2_ or α_3_β_3_) (28). The α subunit of RHDs contains a Rieske [2Fe-2S] center that transfer electrons to iron at the active site, while the β subunit is thought to play a structural role in the enzyme complex (28). We also detected expansion of RHDs in group III and IV members, albeit to a lesser extent than that in group VII (Fig. 2B).

Of the 365 RHD α subunits found in SAR202, 135 copies came from group VII. Group VII genomes had from 1 to 62 paralogous copies of subunit α (COG4638) and 1 to 3 copies of subunit β (COG5517). Given that there are more α than β subunits, it appears that most of the RHDs in group VII function as monomeric RHDs. The highest relative RHD abundance per genome of 2.64% (50 copies) was actually identified in a group IV SAR202 member (OSU_TB11) (Fig. 2B). This is the only member of this group that encodes such a high number of copies of RHDs in its genome.

A sponge symbiont member of group VII (MPMJ01) (18) encodes the largest number of copies of RHDs (62 copies and 1.96% of its genes), but it also has one of the largest genomes at 3.22 Mbp. Most of the RHDs were annotated as phthalate 4,5-dioxygenase oxygenase subunit (38.9% of all annotations), phenoxybenzoate dioxygenase subunit alpha (26%), 3-phenylpropionate/cinnamic acid dioxygenase subunit alpha (20.5%), or carbazole 1,9a-dioxygenase, terminal oxygenase component (8.2%).

While the vast majority of the RHDs are annotated as phthalate 4,5-dioxygenases, it is unlikely that phthalates are common substrates in the ocean. Most group VII MAGs were recovered from euphotic zone samples; all bins originated from ≤200 m depth. OSU_TB11, a group IV SAR202 member, originated in the mesopelagic zone (790 m). We speculate that these enzymes are used to metabolize other monocyclic or polycyclic aromatic compounds released by phytoplankton, providing SAR202 cells with energy and carbon.

A recent paper showed that some SAR202 members encode large numbers of RHDs, which were likely acquired by horizontal gene transfer (HGT), in their genomes and speculated that these play a role in the catabolism of resistant DOM of terrestrial origin (12). In coastal regions of the Indian Ocean, the Red Sea, and the Southern Ocean near Antarctica, we found a large number of MAGs with a high number of paralogous copies of RHDs (Fig. S1), which appeared to support the findings by Colatriano et al. (12).

10.1128/mBio.02975-19.1 FIG S1 (A) Distribution of SAR202 single amplified genomes (SAGs) and metagenome-assembled genomes (MAGs) encoding ring-hydroxylating dioxygenases (RHDs) and (B) SAR202-specific RHD abundances in *Tara* Oceans metagenomes. SAGs/MAGs with highest RHD abundances are located in coastal locations. Samples were normalized by dividing the total number of SAR202 RHDs by the total number of SAR202 single-copy genes found in each sample. Download FIG S1, PDF file, 0.5 MB. Copyright © 2019 Saw et al. 2019 Saw et al. This content is distributed under the terms of the Creative Commons Attribution 4.0 International license.

### Rhodopsins in epipelagic group I and II SAR202 members.

Twenty-eight genomes, all from samples obtained from water depths shallower than 150 m, encoded proteorhodopsins, one of which was a heliorhodopsin. Most of the type 1 rhodopsins were found in members of groups Ia, Ib, Ic, and II, which we report below are prevalent in the euphotic zone. The single heliorhodopsin, which was found in a group II genome, is related to a recently described group of heliorhodopsins (29). Using the backbone tree from that study (29), the SAR202 type 1 rhodopsins were placed close to previously known proteorhodopsins, and the sole heliorhodopsin was placed deep within the newly described heliorhodopsins (Fig. S2 and S3).

10.1128/mBio.02975-19.2 FIG S2 Maximum likelihood phylogenetic tree of rhodopsins found in SAR202 groups, based on a tree from a recent study (29). SAR202 rhodopsins are closely related to blue and green light-absorbing proteorhodopsins (PR). Orange and white node circles indicate ultrafast bootstrap support values above and below 90, respectively. Download FIG S2, PDF file, 0.03 MB. Copyright © 2019 Saw et al. 2019 Saw et al. This content is distributed under the terms of the Creative Commons Attribution 4.0 International license.

10.1128/mBio.02975-19.3 FIG S3 Detailed phylogenetic tree of SAR202 rhodopsins from Fig. S2, showing tips colored according to SAR202 subgroups. The phylogenetic tree was built using IQ-Tree with the following parameters: -m LG+C10+F+G -bb 1000. Download FIG S3, PDF file, 0.04 MB. Copyright © 2019 Saw et al. 2019 Saw et al. This content is distributed under the terms of the Creative Commons Attribution 4.0 International license.

### Depth stratification and biogeography indicate that niche specialization is correlated with expansions of paralogous gene superfamilies in SAR202.

**FIG 5:**
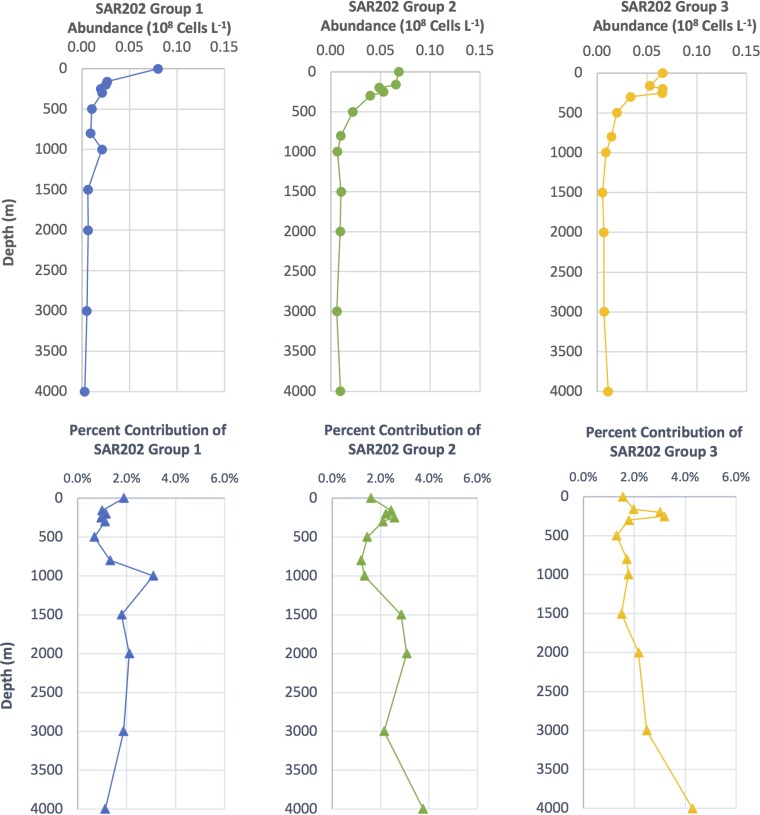
Depth profiles showing SAR202 group I abundance (blue circle and line), SAR202 group II abundance (green circle and line), and SAR202 group III abundance (yellow circle and line) as determined by FISH group-specific oligonucleotide probes. Depth profiles showing SAR202 group I percent contribution to total bacterioplankton determined by 4′,6-diamidino-2-phenylindole (DAPI) cell counts (blue triangle and line), SAR202 group II percent contribution to total bacterioplankton (green triangle and line), and SAR202 group III percent contribution to total bacterioplankton (yellow triangle and line).

**FIG 6:**
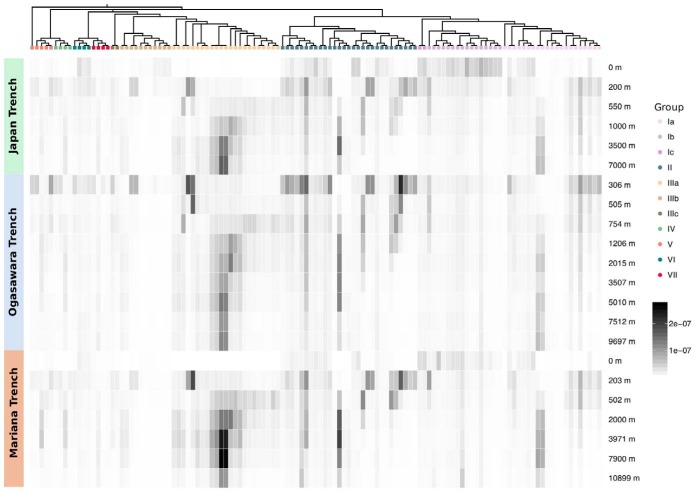
Fragment recruitment analysis of metagenomic reads from three deep ocean trenches against the SAR202 genomes. Arrangement of SAR202 genomes follows the branching order in the Bayesian phylogenomic tree shown in Fig. 1. Recruitment is calculated as the number of bases of metagenomic reads aligned against SAGs or MAGs normalized by the total number of bases present in a given metagenomic sample. The intensity of shading represents the degree of recruitment.

Group I genomes, including those that encoded rhodopsins, mainly originated from epipelagic samples (0 to 200 m), whereas the group III members were mainly retrieved from the mesopelagic zone (200 to 1,000 m) (Fig. 2). FISH and fragment recruitment data confirmed that the major SAR202 subclades show different trends with depth (Fig. 5 and 6). Oceanic water columns have pronounced vertical gradients in light (photosynthetically available radiation [PAR]), inorganic nutrients, and organic matter composition, which likely establish specialized nutritional niches. The vertical stratification of some SAR202 subclades and the evidence described above for metabolic specialization suggest that SAR202 diversity arose through adaptations that enabled the subclades to specialize in resources that vary across the water column.

### Fragment recruitment analyses.

Metagenome fragment recruitment showed that group I members are most abundant in the epipelagic zone (from surface to 200 m); group III recruited more reads from mesopelagic, bathypelagic, abyssopelagic, and hadalpelagic samples, and group II recruited reads from the surface through the mesopelagic zone (Fig. 6, and Fig. S4). In *Tara* Oceans metagenomes, group I members, most notably Ib members, were relatively more abundant in the epipelagic zone (5 to 80 m in the Indian Ocean, 5 to 60 m in the Mediterranean Sea, 100 to 150 m in the South Atlantic Ocean, and 115 to 188 m in the South Pacific Ocean) (Fig. S4A). However, despite decreasing with depth, populations persisted in the deep ocean. In waters overlying the Japan and Mariana Trenches, group I members (particularly Ib members) were abundant only near the surface.

10.1128/mBio.02975-19.4 FIG S4 (A) Fragment recruitment of metagenomic reads from *Tara* Oceans metagenomic samples against all SAR202 SAGs and MAGs. Color boxes on the left of the heatmap represent different oceanic regions, with the abbreviations of these oceanic regions shown in the boxes. Metagenomic samples are arranged according to depth, and sample names and depth information are shown on the right of the heatmap. Branching order of the SAR202 genomes follow the orders shown in the Bayesian phylogenetic tree in Fig. 1. (B) Fragment recruitment of metagenomic reads from BATS metagenomic samples against all SAR202 SAGs and MAGs. Color boxes on the left of the heatmap represent different depths, and depth information is shown in the box. Metagenomic samples are arranged according to depth, and sample names are shown on the right of the heatmap. Branching order of the SAR202 genomes follows the order shown in the Bayesian phylogenetic tree in Fig. 1. Download FIG S4, PDF file, 0.1 MB. Copyright © 2019 Saw et al. 2019 Saw et al. This content is distributed under the terms of the Creative Commons Attribution 4.0 International license.

There is a noticeable absence of group IIIa members in the upper water column shallower than 200 m in the metagenomes from the Northwestern trench (Fig. 6), shallower than 250 m in the *Tara* Oceans metagenomes (Fig. S4A), and shallower than 200 m in the BATS metagenomes (Fig. S4B). Group IIIa SAR202 are most abundant in deep ocean regions (600 to 1,000 m in the Indian Ocean, 590 to 800 m in the North Atlantic Ocean, 700 to 800 m in the South Atlantic Ocean, 375 to 650 m in the North Pacific Ocean, 350 to 696 m in the South Pacific Ocean, and 790 m in the Southern Ocean) (Fig. S4A). Group IIIa members are found almost exclusively deeper than 200 m (200 to 7,000 m in the Japan Trench, 306 to 9,697 m in the Ogasawara Trench, and 203 to 10,899 m in the Mariana Trench). Members of group IIIb, however, appear to be more abundant in the upper water columns and less so in the deeper zones in two metagenomic data sets (Fig. 6 and Fig. S4A).

Group II members seem to occupy transitional zones between those occupied by group I and group III members (for example, 270 to 600 m in the Indian Ocean, 250 m in the North Atlantic Ocean, and 40 to 450 m in the North Pacific Ocean). However, the depth zones occupied by group II members also seem to largely overlap those of both group I and group III members (Fig. 6 and Fig. S4A). Group II members are again found to occupy intermediate depths in the Northwestern Pacific Ocean trenches (200 to 1,000 m at Japan Trench, 306 to 1,206 at Ogasawara Trench, and 203 to 502 m at Mariana Trench). Some group II members are found over wider depth ranges, with one quite abundant in the deepest water samples in all three trenches (Fig. 6).

### Group I, II, and III fluorescent *in situ* hybridization profiles.

Separate oligonucleotide probes were designed in this study for SAR202 groups I, II, and III (Data Set S1). A FISH protocol was optimized for each of the probes by experimentally determining dissociation curves to select washing temperatures (Fig. S8A). The SAR202 group I probe had the highest fluorescent intensity at 52.5°C. The SAR202 group II probe had the highest intensity at 55°C, and the SAR202 group III probe had the highest intensity at 57.5°C. All three groups consisted of small round cells (Fig. S8B). Group III cells were the largest, with a diameter of 1 μm (Fig. S8B).

10.1128/mBio.02975-19.8 FIG S8 (A) Dissociation curves for SAR202 group I (blue line), SAR202 group II (green line), and SAR202 group III (yellow line) probes with washing temperature (°C) on the *x* axis and average intensity of the probe on the *y* axis. (B) Black and white overlaid images showing the SAR202 cell morphology for groups I, II, and III. Cy3 images were segmented with Image Pro Plus software v 7.0 (Media Cybernetics, Bethesda, MD, USA) and overlaid onto corresponding segmented 4′,6-diamidino-2-phenylindole (DAPI) images. Objects with overlapping signals in both Cy3 and DAPI images were counted as probe positive. Cells that were positive for both Cy3 and DAPI are white, while cells that are positive with either Cy3 or DAPI are grey. The positive cells are marked with blue arrows for group I, green arrows for group II, and yellow arrows for group III. Group I cells are shown from 1,000 m, group II cells are shown from 250 m, and group III cells are shown from 4,000 m. Larger scale bars represent 10 μm and smaller scale bars represent 1 μm in each of the images. Download FIG S8, PDF file, 1.4 MB. Copyright © 2019 Saw et al. 2019 Saw et al. This content is distributed under the terms of the Creative Commons Attribution 4.0 International license.

The group-specific oligonucleotide probes for SAR202 groups I, II, and III were developed and used to count cells throughout the BATS water column to 4,000 m in July 2017 (Fig. 5). All three groups were detected in significant numbers throughout the water column, summing to about 5% of total bacteria near the surface and up to 10% at 4,000 m. Group I SAR202 cell numbers peaked in the epipelagic zone and dropped off sharply below the euphotic zone (100 m), whereas groups II and III both had a broader distribution across the epipelagic zone, peaking sharply within the upper mesopelagic zone at ∼250 m, as reported previously. When plotted as relative abundances (Fig. 5, lower panels), the direct cell count data were consistent with the observations from metagenome recruitment, which are also presented in relative units.

### SAR202 FMNO gene relative abundance is correlated with depth.

**FIG 7:**
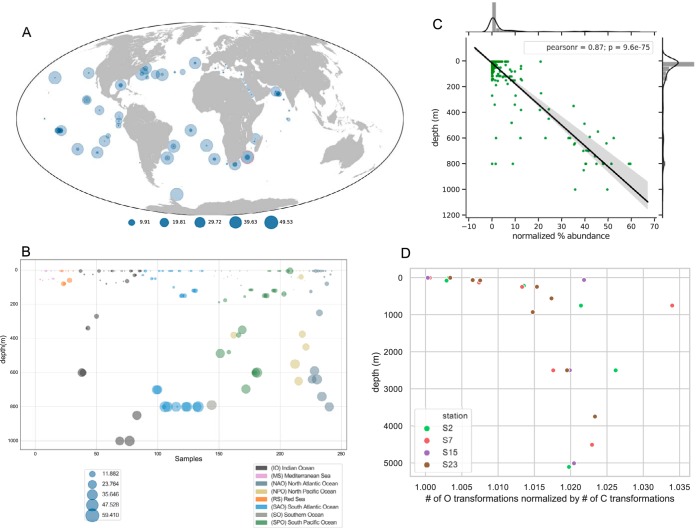
(A) World map showing relative abundances of SAR202-specific FMNOs in *Tara* Oceans metagenomes. Sample with highest relative abundance is highlighted with a red circle. (B) SAR202-specific FMNO relative abundances versus depth in *Tara* Oceans metagenomes. (C) Normalized FMNO abundances in SAR202 are highly correlated with depth in *Tara* Oceans metagenomes. Normalization of FMNO abundances was obtained by dividing the total number of SAR202 FMNOs by the total number of SAR202 single-copy genes found in each sample. (D) The ratio of observations of organic metabolites with mass/charge ratio (*m/z*) that differ in mass by one oxygen to observations that differ in mass by one carbon in Fourier-transform ion cyclotron resonance mass spectrometry (FTICR-MS) data from deep ocean marine DOM samples collected from the Western Atlantic. The stations ranged from 38°S (station 2) to 10°N (station 23). Across the full data set, the most common *m/z* difference observed corresponds to one carbon atom of mass. The data show that transformations corresponding to the addition of a single oxygen atom, as would be catalyzed by a flavin-dependent monooxygenase, become relatively more frequent in the dark ocean. Of several patterns predicted from a previous study (2), this one alone showed a consistent trend.

The relative abundance of all *Tara* FMNO genes (Fig. S7C), and that of SAR202-specific FMNOs, was correlated with depth (Fig. 7C), with Pearson’s *r* values for the latter of 0.87 (*P* = 9.6E^−75^). From these results, it was clear that FMNOs appear to be more functionally important in the deepest ocean regions. Because it appeared that FMNOs are most abundant in SAR202 members originating from the bathypelagic and abyssopelagic zones, we checked to see if the relative abundances of FMNOs in SAR202 genomes correlated with depth. Fig. S5A shows a significant positive correlation between FMNO relative abundance versus depth, and Fig. S5B shows weak but significant negative correlation between enolase abundances versus depth. These data indicate that FMNOs are mostly abundant in SAR202 cells from deep waters, whereas the enolases are more abundant in shallow water ecotypes.

10.1128/mBio.02975-19.5 FIG S5 Correlation of relative enzyme abundances versus depth of origin of the most abundant paralogous families of genes in SAR202 SAGs and MAGs. The enzyme families are (A) flavin-dependent monooxygenases (FMNOs), (B) enolases, (C) RHDs, and (D) dehydrogenases. Download FIG S5, PDF file, 0.5 MB. Copyright © 2019 Saw et al. 2019 Saw et al. This content is distributed under the terms of the Creative Commons Attribution 4.0 International license.

10.1128/mBio.02975-19.7 FIG S7 (A) World map showing relative abundances of all FMNOs identified in all *Tara* Oceans metagenomes. These include SAR202-specific FMNOs and those from other organisms. The sample with highest relative abundance is highlighted in red. Different sizes of the bubbles represent the different percentages of abundance as shown in the circles below the map. (B) Relative abundances of FMNOs along depth profile in all *Tara* Oceans metagenomes. Samples are sorted in order of sampling time (from beginning to end). (C) Correlation between relative abundances of all FMNOs in *Tara* metagenomes versus depth. Download FIG S7, PDF file, 2.0 MB. Copyright © 2019 Saw et al. 2019 Saw et al. This content is distributed under the terms of the Creative Commons Attribution 4.0 International license.

We used ultrahigh resolution mass spectrometry to characterize the composition of dissolved organic matter in seawater from the western Atlantic Ocean. The resolution of the mass spectrometer allows us to assign differences in measured mass/charge (*m/z*) values based on the gain or loss of elements such as carbon, hydrogen, oxygen, or other heteroatoms. The analysis shown in Fig. 7D tests the prediction that molecules differing by the addition of a single oxygen atom, as expected from the chemical mechanism of FMNO enzymes, should be more abundant in the deep ocean. In the plot, the ratio of the number of *m/z* observations that differ in mass by one oxygen to the number of *m/z* observations that differ in mass by one carbon increases dramatically below the epipelagic zone. In the model we presented previously, cells are presumed to enzymatically modify recalcitrant DOM compounds, channeling some to catabolism and exporting the remainder that cannot be degraded out of the cell (2).

### Enolase abundances show weak correlation with depth.

Because enolases appear to be a notable feature of SAR202 SAGs and MAGs from the upper water column, we assessed whether relative enolase abundances were also correlated with depth. Figure S5B shows that there is a slight negative correlation between the percent abundance of enolase genes in MAGs and SAGs and the depth from which they were recovered, but those of SAR202 enolases in the *Tara* Oceans metagenomic data are positively correlated with depth (Pearson’s *r* value, 0.6; *P* = 1.4E^−25^) (Fig. S6). This was surprising because we reasoned, based on the genomic data, that the enolases might be involved in breaking down more labile compounds found in the upper water column and expected higher abundances of enolases in the *Tara* Oceans samples from upper water columns. One explanation for this discrepancy could be biased sampling of MAGs from the *Tara* Oceans metagenome samples. We selected 43 *Tara* samples to assemble based on SAR202 abundances; some samples from deeper regions that we did not assemble could harbor uncharacterized SAR202 subgroups.

10.1128/mBio.02975-19.6 FIG S6 (A) Relative abundances of SAR202-specific enolases in *Tara* Oceans metagenome samples. Distribution of samples is plotted in order of sampling dates and depth of origin of the samples. (B) Correlation of normalized SAR202-specific enolase relative abundances versus depth of origin in *Tara* Oceans metagenome samples. Samples were normalized by dividing the total number of SAR202 enolases by the total number of SAR202 single-copy genes found in each sample. Download FIG S6, PDF file, 0.3 MB. Copyright © 2019 Saw et al. 2019 Saw et al. This content is distributed under the terms of the Creative Commons Attribution 4.0 International license.

## DISCUSSION

Pangenome analysis confirmed earlier reports of ancient expansions of paralogous enzymes in the SAR202 clade (Fig. 2B and 4A and 4B). The paralogous gene families were correlated with deep branches in the SAR202 genome tree that divide the clade into seven subgroups. Metagenome analyses, and cell counts made with FISH probes, showed that several of the SAR202 groups are vertically stratified, suggesting niche specialization (Fig. 5 and 6). Collectively, these patterns amount to strong evidence that the early evolutionary radiation of SAR202 into subgroups was accompanied by metabolic specialization and expansion into different ocean niches.

It is striking that the three major paralog expansions in SAR202 suggest three different metabolic strategies, each of which could be interpreted as an adaptation to metabolizing a class of recalcitrant organic compounds. These hypothetical schemes assume that the evolutionary diversification of paralogous enzyme families was driven by selection favoring substrate range expansion. We found support for these schemes in evidence that these gene lineages arose early in evolution. While deep internal nodes for these genes in tree topologies could result from the recruitment of paralogs by horizontal gene transfer, the rarity of near-gene neighbors across the tree of life favors the explanation that most of the paralog diversity arose within SAR202 by gene duplication during evolution. If this interpretation is correct, it implies that much of the functional diversity in two major enzyme families, the alkanal monooxygenases within the FMNO superfamily and the madelate racemases within the racemase superfamily, may have originated within SAR202. This is apparently not the case for group VII and IV dioxygenases, for which there is evidence of acquisition by HGT (12).

Surprisingly, because SAR202 members have the reputation of being deep ocean microbes, the ecological data we gathered revealed that group I SAR202 members are mainly epipelagic and that they harbor large and diverse families of enolase paralogs. Enolase superfamily enzymes remove the α-proton from carboxylic acids to form enolic intermediates, which can rotate on the axis of the double bond of the intermediate, with stereochemical consequences (20). These enzymes catalyze racemizations, β-eliminations of water, β-eliminations of ammonia, and cycloisomerizations. Chemical oceanographers have recognized a role for molecular chirality in diagenesis, reporting that the ratio of d-aspartic to l-aspartic acid uptake by prokaryotic plankton increases by two to three orders of magnitude between surface and deep mesopelagic waters in the North Atlantic (30). This has been interpreted as evidence that mesopelagic prokaryotic plankton are using bacterial cell wall-derived organic matter because the bacterial peptidoglycan layer is the only major biotic source of significant d-amino acids in the ocean (31). However, information about d-amino acid utilization by marine microbes remains limited (32).

We propose that chiral complexity defines a class of resistant compounds, and that enolases are an innovation that makes this DOM accessible to degradation by reducing the number of enzymes needed to degrade it. The number of enantiomers of a compound increases by 2*^n^*, where *n* is the number of chiral centers. To illustrate, a single compound with three chiral centers might in principle require eight enzymes to recognize all stereoisomers. However, if the three chiral centers were racemized by enolases, then only four enzymes would be required—one degradative enzyme and one enzyme to racemize each of the chiral centers. Spontaneous racemization might play a role in increasing the chiral complexity of DOM and thereby transitioning it to more resistant forms, but chiral complexity might also originate from biocomplexity, such as diversity in cell wall composition, much of which is unexplored. An alternative hypothesis that could also explain our findings is the use of enolases to synthesize chirally complex organic matter, but at present there is no precedent that establishes a useful purpose for such metabolism at the scale of the paralog expansion we report. β-Eliminations, another possibility for enzymes in this family, would essentially serve the same functions as racemases in catabolic contexts, yielding dehydrated ketone products from enantiomeric mixtures.

Group II SAR202 members, which are most abundant in the mesopelagic, maintain both the enolase and FMNO enzyme families in equal abundances, suggesting they use both DOM resources—chirally complex organic matter and compounds that can be catabolized via monooxygenases—in this intermediate water column zone. Earlier studies have demonstrated that, in addition to a dissolved organic carbon (DOC) concentration decreasing with ocean depth, the abundance of diagenetically altered DOM compounds increases below the euphotic zone (33–35). In bathypelagic, abyssopelagic, and hadalpelagic regions, group III species dominate, presumably indicating that molecules susceptible to oxidation by FMNOs become one of the few remaining harvestable DOM resources at these depths. Close examination of Fig. 6 shows that there are more finely structured patterns of congruence between tree topologies and depth range than the broad patterns on which we focus our discussion. For example, some lineages of group Ia were consistently observed in the bathypelagic zone, and some group II members were observed near the surface. It is apparent that more complex relationships between ecology, evolution, and metabolism remain to be explored in SAR202.

This study confirmed previous reports of expansions of FMNO enzymes in group III genomes recovered from the deepest ocean regions (2) and of RHD enzymes in genomes from coastal sites. Both FMNO and RHD enzymes are powerful oxidases implicated in the catabolism of resistant compounds, such as sterols and lignins. Addition of lignin, a model recalcitrant compound, has been shown in a recent study to stimulate SAR202 growth (36). The expansion of these enzyme families is proposed to have enabled SAR202 to exploit new niches defined by these DOM resources. In the case of group IV members, this would be lignins and other aromatic compounds of terrestrial origin, whereas group III members are proposed to partially oxidize a wide variety of recalcitrant molecules, perhaps including sulfonates and heterocyclic compounds. It has been hypothesized that the partial oxidation of these compounds might produce more recalcitrant compounds that accumulate (2).

The genome-enabled hypotheses we propose are challenging to test, but nonetheless should be studied because the tremendous size of the organic carbon pool in question. Deep ocean regions beyond the reach of sunlight contain an estimated 662 Pg of DOC (37), which ranges in quality between labile DOM and RDOM (38, 39). If our hypotheses are correct, this pool would be much larger if cells had not evolved strategies to oxidize many forms of resistant DOM. In principle, the modern refractory DOM pool would become much smaller if contemporary cells evolved mechanisms to oxidize it, with catastrophic consequences for the environment.

The complexity of ocean DOM presents many challenges to proving these hypotheses. An example of these problems is the issue of chemical enantiomers, which have identical empirical formulas that make them unresolvable by mass. With respect to other hypotheses we advance, DOM chemical structural diversity has not been described with sufficient resolution to support a comprehensive accounting of the compounds present in DOM, which complicates the problem of selecting compounds for empirical tests with live cells. Moreover, we have no reason to think that contemporary knowledge of enzyme activities and metabolic processes in bacteria is complete and sufficient to provide a basis for thorough models for carbon oxidation and sequestration. In brainstorming these challenges, we encountered successes in confirming a pattern in DOM *m/z* spectra that fits one of our hypotheses (Fig. 7D), and we also identified at least one recalcitrant compound that stimulates SAR202 cell growth (36). Ongoing work in our laboratories is focused on further functional studies that test the metabolic models we propose here. It is our contention that such work is needed to expand knowledge of the biochemistry and cell biology of microorganisms that transact carbon cycle processes in the oceans.

## MATERIALS AND METHODS

Methods for metagenomic library preparation and sequencing, single-gene phylogenetic and phylogenomic analyses, direct cell counts and fluorescent *in situ* hybridization of SAR202, mass spectrometry, and a detailed proposal to name SAR202 groups can be found in the supplemental material.

### Sample collection and sequencing of single amplified genomes and shotgun metagenomic sequencing from the three trench sites.

SAG generation was performed using fluorescence-activated cell sorting and multiple displacement amplification at Bigelow Laboratory Single Cell Genomics Center (SCGC; scgc.bigelow.org), as previously described (40). Selection for genomic sequencing was aimed at representing the diverse SAR202 subgroups based on their 16S rRNA phylogenetic tree placement, and 10 single-cell amplified genomes (SAGs) were selected for genomic sequencing based on their phylogenetic placement (data not shown). They originate from samples from three deep sea trenches in the Northwestern Pacific Ocean: the Mariana, Japan, and Ogasawara Trenches. Water samples from the central part of the Izu-Ogasawara (Izu-Bonin) Trench (29°9.00′N, 142°48.07′E; 9,776 m below sea surface [mbs]) were obtained using Niskin-X bottles (5-liter type, General Oceanics) during a total of two dives of the ROV *ABISMO* during the Japan Agency for Marine-Earth Science and Technology (JAMSTEC) R/V *Kairei* KR11-11 cruise (December 2011). Water samples from the southern part of the Japan Trench (36°5.88′N, 142°45.91′E; 8,012 mbs) was obtained by vertical hydrocasts of the conductivity temperature depth profiler with carousel multiple sampling system (CTD-CMS) with Niskin-X bottles (12-liter type, General Oceanics) during the JAMSTEC R/V *Kairei* KR12-19 cruise (December 2012). From the Challenger Deep of the Mariana Trench, water samples (except for the trench bottom water) were taken by Niskin-X bottles (5-liter type) on the ROV *ABISMO*, and the trench bottom water was obtained by a lander system (41) during the JAMSTEC R/V *Kairei* KR14-01 cruise (January 2014). Samples for SAG generation were stored at −80°C with 5% glycerol and 1× Tris-EDTA buffer (final concentrations) (42). For the shotgun metagenomic library construction, microbial cells in approximately 3 to 4 liters of seawater were filtered using a cellulose acetate membrane filter (pore size, 0.22 μm; diameter, 47 mm; Advantec, Tokyo, Japan).

Four SAGs were sequenced at SCGC, and six SAGs were sequenced at the Center for Genome Research and Biocomputing (CGRB) at Oregon State University after Nextera XT sequencing libraries were prepared at JAMSTEC. Sequencing libraries for SAGs obtained from the Mariana Trench site was directly synthesized with a Nextera XT DNA library preparation kit as described previously (43). The number of amplification cycles for the construction of these libraries was 17, except the case of AD AD-812-D07, which had 12 cycles of amplification.

### Genome assemblies, binning, and annotation.

Illumina library preparation, sequencing, *de novo* assembly, and quality checking of SAGs AC-409-J13, AC-647-N09, AC-647-P02, and AD-493-K16 were performed by SCGC, as previously described (40). For the remaining six SAGs, raw sequences were first quality trimmed using Trimmomatic (44). Four SAGs were assembled individually using SPAdes assembler version 3.9.0 (45) with “–careful and –sc” flags. Due to cross-contamination present in a second batch of 6 SAGs sequenced, they were coassembled using metaSPAdes, and then CONCOCT was used to separate the contigs from each SAG into respective bins. CheckM analysis of the bins showed that contamination levels in each identified bin were very low (below 0.2%) and that the 6 SAGs are from very divergent clades, so they can be easily separated by a differential coverage binning approach.

Raw sequences from 17 metagenomics samples from the Bermuda Atlantic Time-series Study (BATS) and 43 metagenomic samples from the *Tara* Oceans expedition were quality trimmed using Trimmomatic and individually assembled using metaSPAdes version 3.9.0 (46). The 43 *Tara* Oceans metagenomes chosen contain at least 1% relative SAR202 abundance based on metagenomics tag (miTAG) sequence data (47) (Data Set S1).

All metagenomics contigs larger than 1.5 kbp were separated using MetaBAT (48) to gather potential SAR202 bins. MetaBAT requires the use of multiple samples to calculate contig abundance profile in the samples. For *Tara* Oceans metagenomes, in order to generate abundance profiles, contigs were mapped against a minimum of 10 *Tara* Oceans metagenome samples chosen randomly (including the sample from which the contigs were assembled) using BBmap (http://sourceforge.net/projects/bbmap/). For BATS metagenomes, BBmap was also used against all 17 metagenomes to generate contig abundance profiles. Identities of the resulting bins were checked for presence of 16S rRNA gene sequences matching known SAR202 sequences from Silva database release 128. In cases where there were no 16S rRNA genes in the bins, concatenated ribosomal protein phylogenies were constructed to identify members of the SAR202 clade. A total of 26 MAGs from a recent study (19) were also included in the binning process. These were also metagenomic bins from *Tara* metagenomes assembled with MEGAHIT. The list of bins used in this study is shown in Data Set S1. We also checked the bins obtained by another study using the *Tara* metagenomes (17) to see if there are redundant genome bins in our assemblies.

After potentially novel SAR202 bins were identified, average nucleotide identities between all *Tara* genome bins were determined with the PyANI tool (https://github.com/widdowquinn/pyani), and a custom Python script “osu_uniquefy_TARA_bins.py” was used to identify bins that share 99% average nucleotide identity (ANI). When nearly identical bins were matched, the more complete and less contaminated genome bin was retained. In cases where bins originated from the same *Tara* station, near-identical bins were combined and coassembled with Minimus2 (49) to improve the genome completeness. Refinement of metagenomic bins was done using the Anvi’o tool (50) to identify any potentially contaminating contigs. Some genomic bins were entirely discarded if too many multiple copies of single-copy genes are present that could not be separated by Anvi’o. Genome completeness and redundancies were estimated using the CheckM tool (51). Genomes at various levels of completion with less than 1.1% redundancy of single-cope marker genes and less than 5% contamination were included for further analyses.

All the SAGs and MAGs were annotated with Prokka version 1.11 (52) to assign functions. Coding sequences predicted by Prokka were also submitted to the GhostKOALA web server (53) to assign Kyoto Encyclopedia of Genes and Genomes (KEGG) annotations to the predicted genes. In addition, InterProScan (database version 5.28-67.0) and eggNOG-mapper (54) searches were also carried out. Metagenome-assembled genomes (MAGs) and SAGs from previous studies were also reannotated together with the new genomes to keep the functional assignments consistent.

### Metagenome fragment recruitment analyses.

Recruitment of quality-trimmed metagenomic reads from three different metagenomic databases against the SAG and MAG contigs masked to exclude rRNA-coding regions (16S, 23S, and 5S rRNA genes as predicted by barrnap) was done using FR-HIT (55) with the following parameters: “-e 1e-5 -r 1 -c 80.” These parameters allowed for reads matching a given reference genome with a similarity score of 80% or higher to be counted as positive matches. The metagenomic samples used for fragment recruitment were 17 samples from BATS, 43 samples from *Tara*, and 22 samples from deep sea trenches (6 from the Japan Trench, 9 from the Ogasawara Trench, and 7 from the Mariana Trench) (Data Set S1). Recruitment was calculated as a percentage of quality-trimmed metagenomic reads aligned against a SAG or a MAG genome size in base pairs, normalized by the total base pairs of reads in a given sample. A recruitment plot was made using the Python script “osu_plot_recruitment_heatmap.py” (see https://bitbucket.org/jimmysaw/sar202_pangenomics/src/master/).

### Analysis of *Tara* Oceans metagenome SAR202 enzyme abundances.

A custom Kraken (56) database was first built from the 122 SAR202 genomes used in this study. All coding DNA sequences in the 243 *Tara* Oceans metagenomic samples were then searched against the custom Kraken database containing SAR202 genomes with rRNA regions masked to identify all coding sequences belonging to SAR202 genomes.

### Data availability.

All of the SAGs and metagenomes are deposited at the National Center for Biotechnology Information (NCBI) and their accession numbers are listed in Data Set S1. Prokka annotations of the genomes are available on figshare (https://doi.org/10.6084/m9.figshare.8343809). All of the metagenomes used for fragment recruitment analysis have been deposited in the DNA Data Bank of Japan under the following submission identifiers: DRA005790 (Ogasawara Trench), DRA005791 (Japan Trench), and DRA005792 (Mariana Trench). Accession numbers of each metagenomic sample are provided in Data Set S1. All code (Bash, Python, and R scripts) used to analyze data and to generate figures are accessible at a Bitbucket repository (https://bitbucket.org/jimmysaw/sar202_pangenomics/src).

10.1128/mBio.02975-19.9 TEXT S1 Text document describing extended methods and naming of SAR202 groups. Download Text S1, DOCX file, 0.03 MB. Copyright © 2019 Saw et al. 2019 Saw et al. This content is distributed under the terms of the Creative Commons Attribution 4.0 International license.
