# Supplementary material for: Pangenomics Analysis Reveals Diversification of Enzyme Families and Niche Specialization in Globally Abundant SAR202 Bacteria
Source: mBio. 2020 Jan 7;11(1):e02975-19. doi: 10.1128/mBio.02975-19 (PMC6946804; doi:10.1128/mBio.02975-19)
Supplement: FIG S1 [file mBio.02975-19-sf001.pdf]

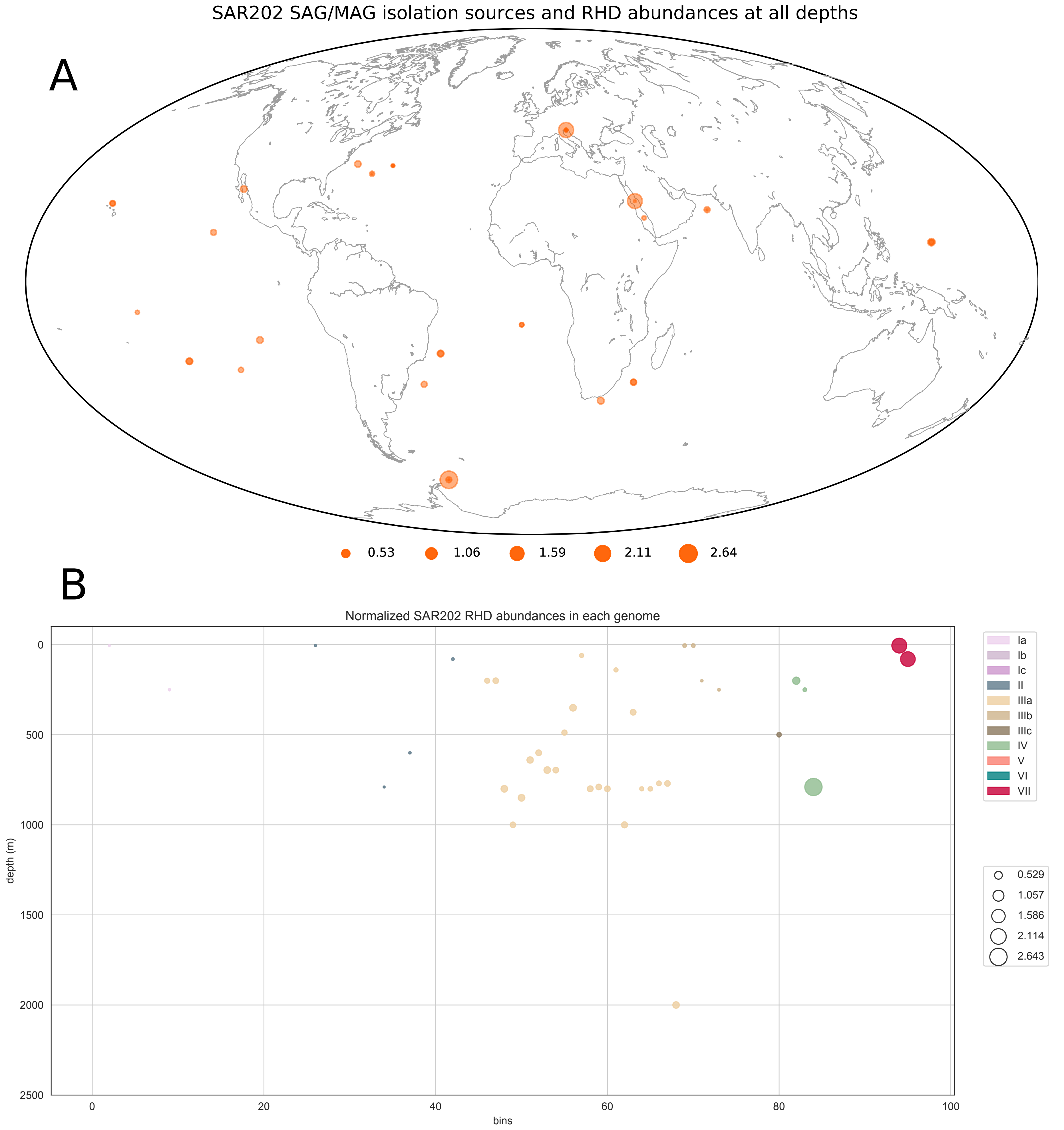
SAR202 SAG/MAG isolation sources and RHD abundances at all depths
A
0.53
1.06
1.59
2.11
2.64
B
Normalized SAR202 RHD abundances in each genome
depth (m)
bins
0
500
1000
1500
2000
2500
0
20
40
60
80
100
Ia
Ib
Ic
II
IIIa
IIIb
IIIc
IV
V
VI
VII
0.529
1.057
1.586
2.114
2.643
