# Supplementary figures and images for: Pangenomics Analysis Reveals Diversification of Enzyme Families and Niche Specialization in Globally Abundant SAR202 Bacteria
Source: mBio. 2020 Jan 7;11(1):e02975-19. doi: 10.1128/mBio.02975-19 (PMC6946804; doi:10.1128/mBio.02975-19)

## Type-1 rhodopsins

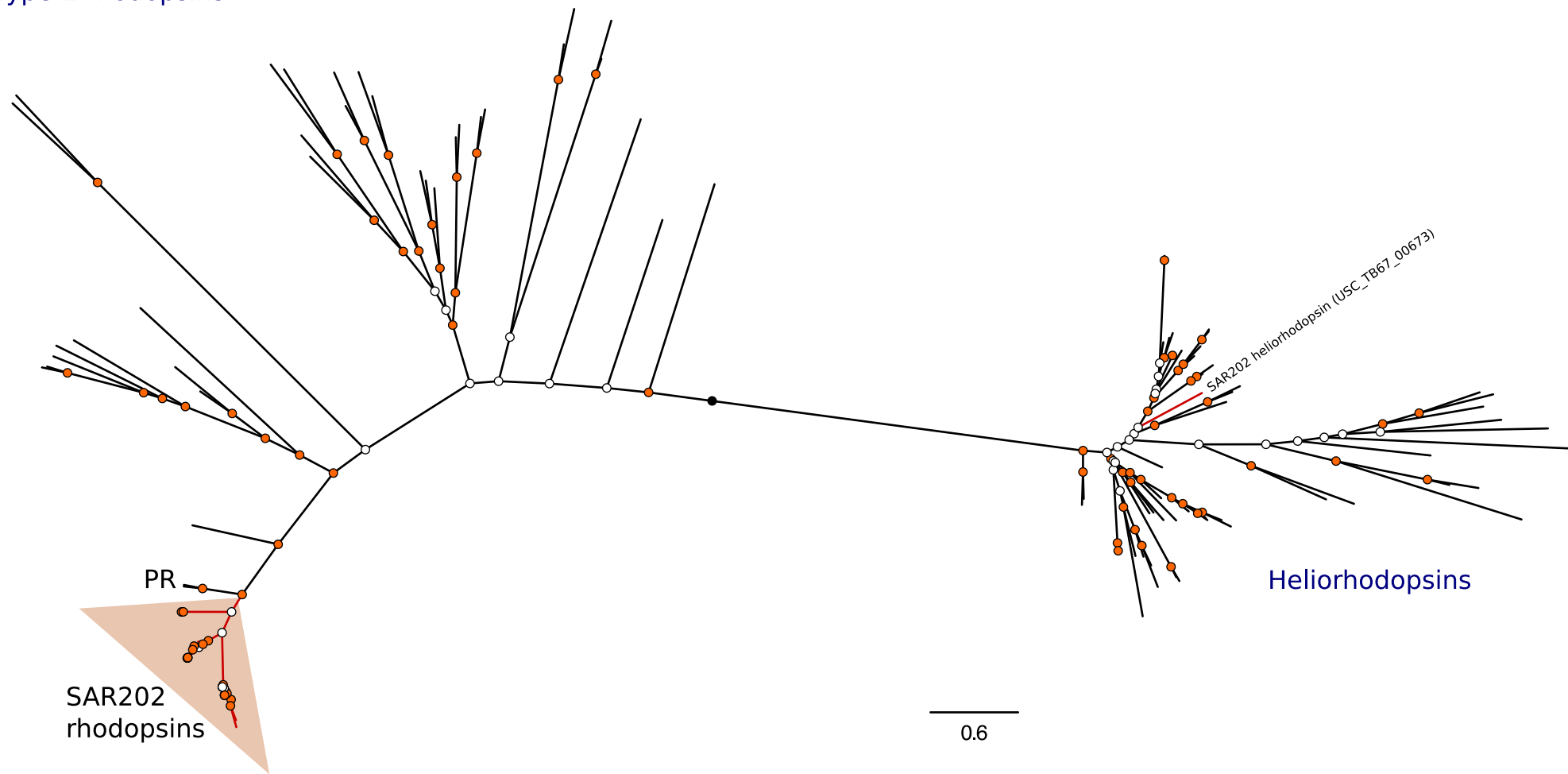

Supplement: FIG S2 [file mBio.02975-19-sf002.pdf]

SAR202  
groups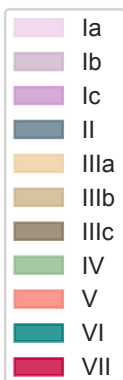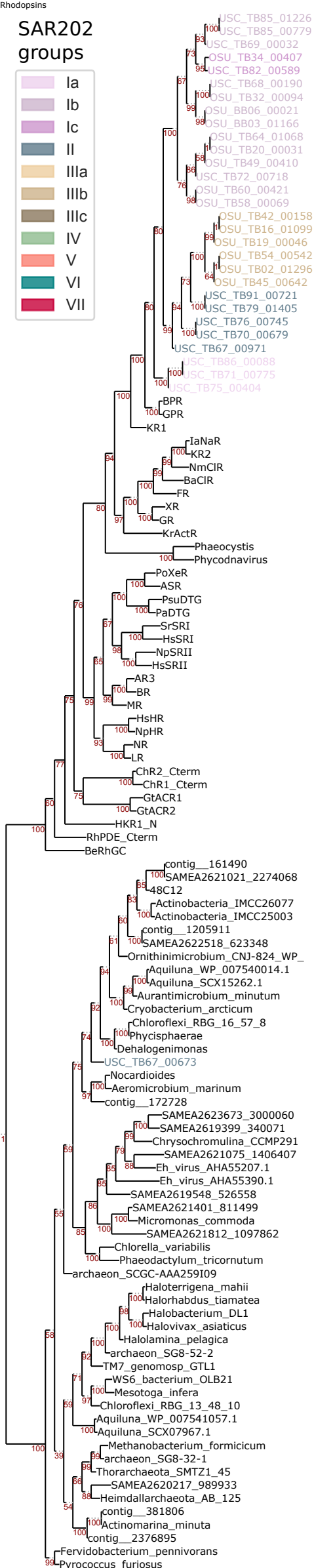

Supplement: FIG S3 [file mBio.02975-19-sf003.pdf]

A

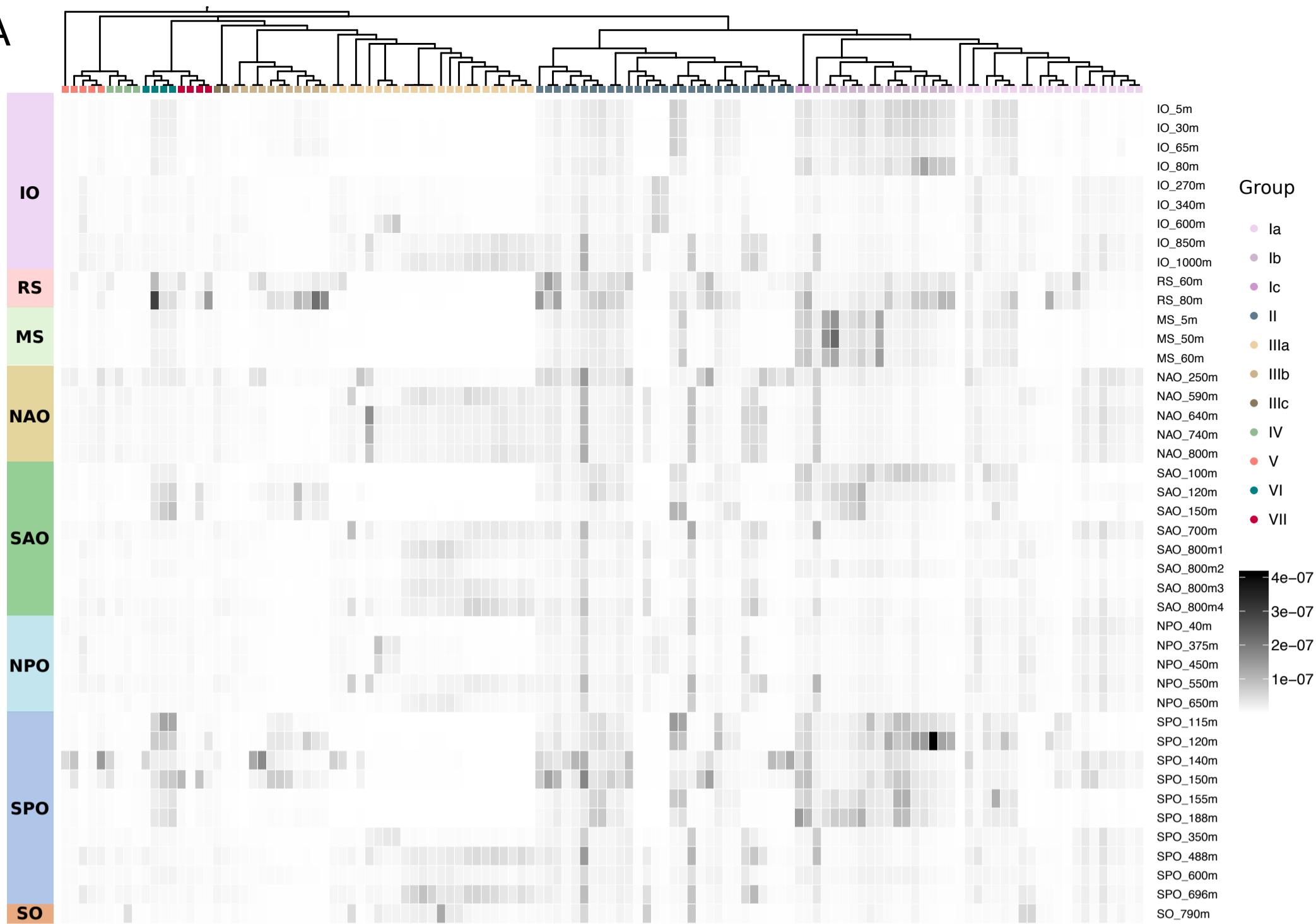

B

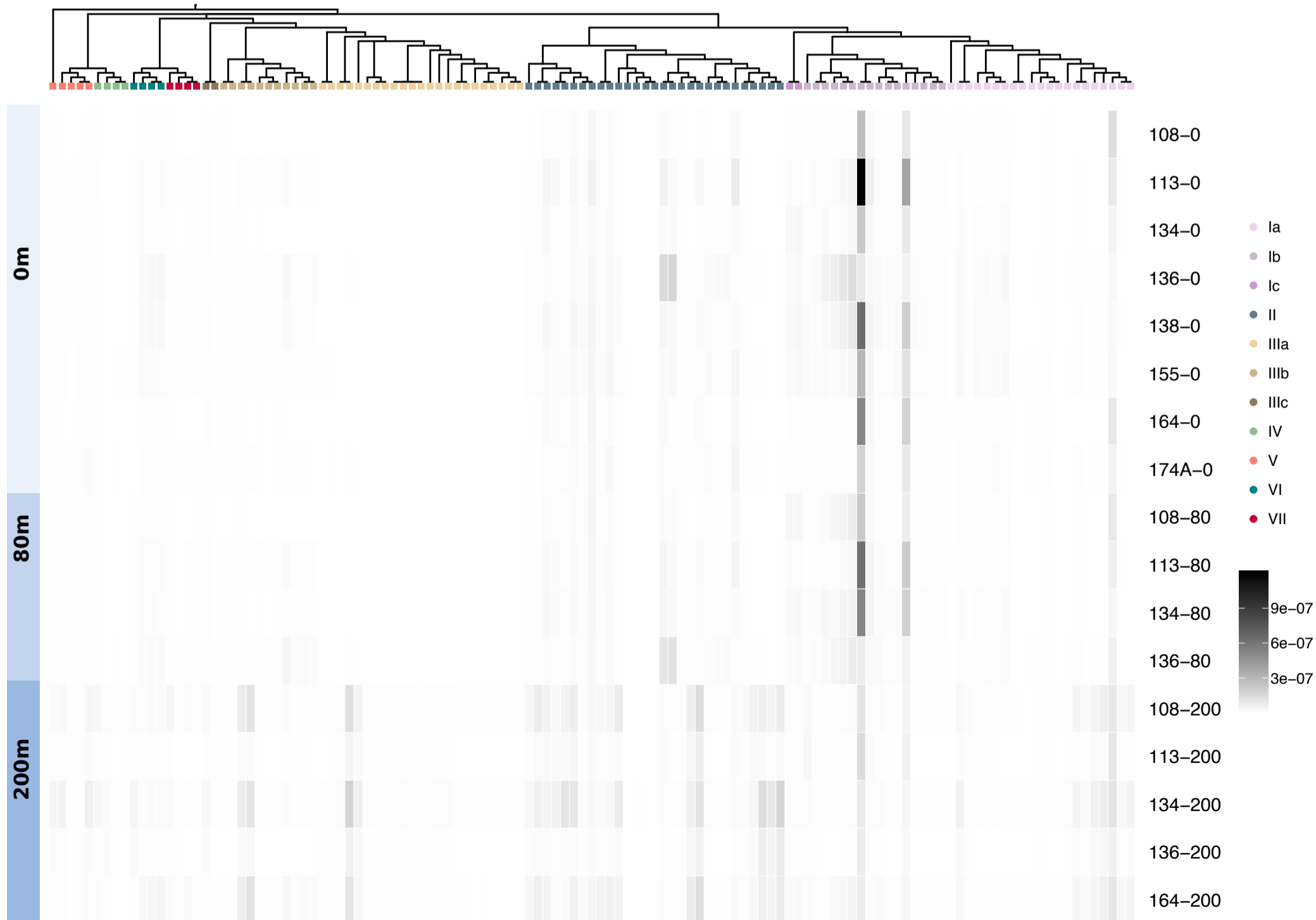

Supplement: FIG S4 [file mBio.02975-19-sf004.pdf]

# A

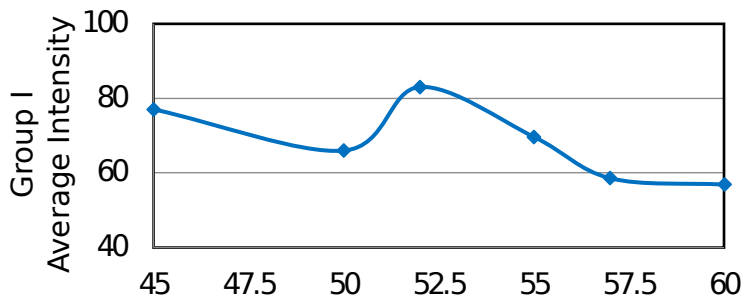

# B

SAR202 Group I

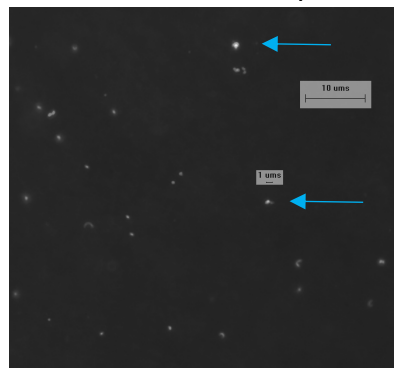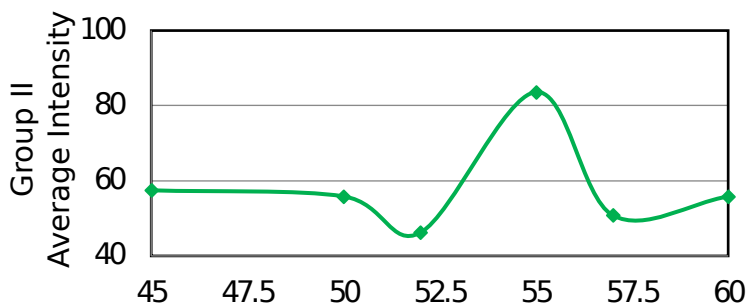

SAR202 Group II

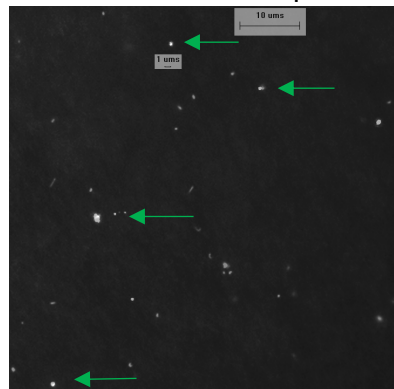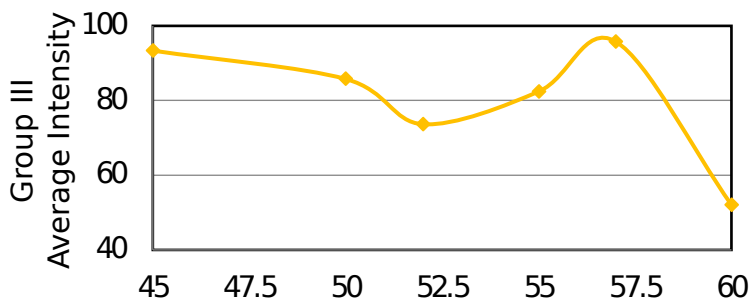

SAR202 Group III

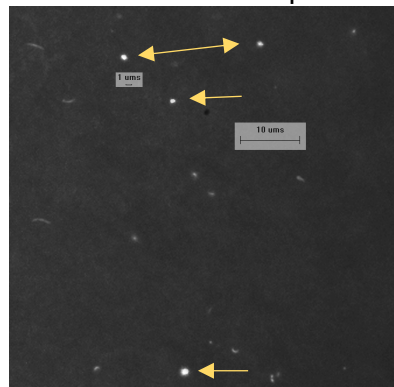

Temperature (°C)

Supplement: FIG S8 [file mBio.02975-19-sf008.pdf]

A

SAR202-specific enolase abundances in TARA Oceans metagenomes

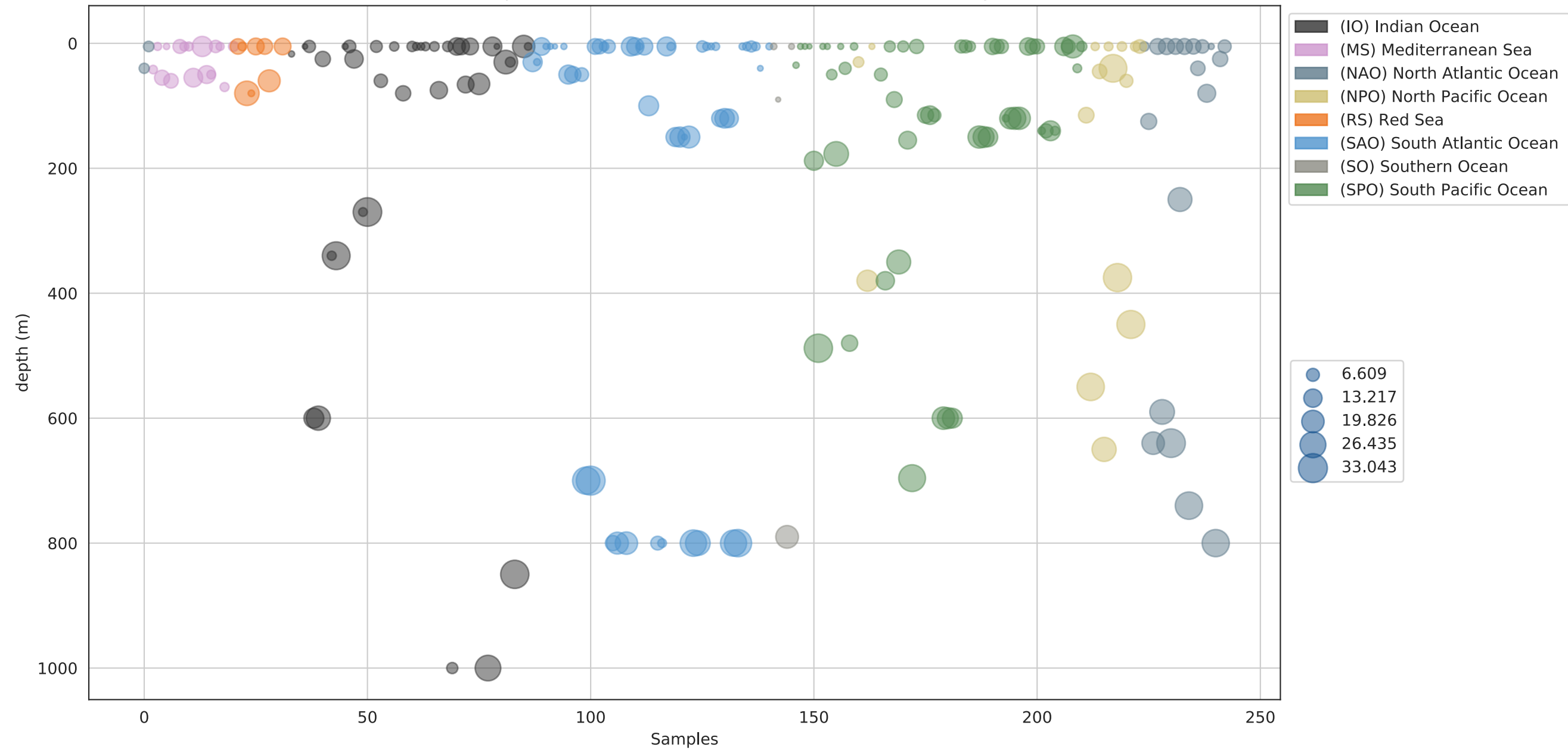

B

%abundance of enolases vs. depth

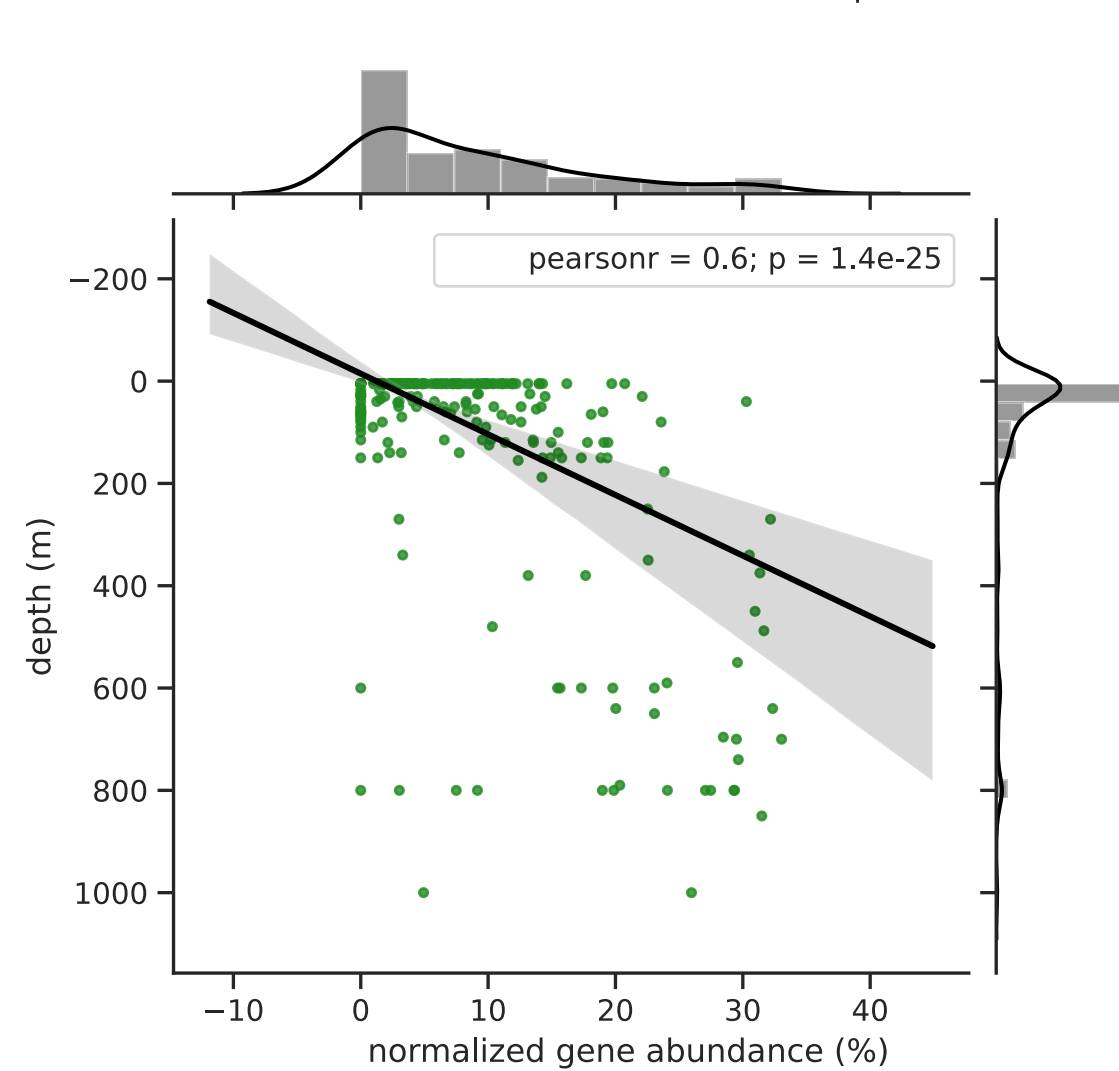

Supplement: FIG S6 [file mBio.02975-19-sf006.pdf]
