# Supplementary material for: Pangenomics Analysis Reveals Diversification of Enzyme Families and Niche Specialization in Globally Abundant SAR202 Bacteria
Source: mBio. 2020 Jan 7;11(1):e02975-19. doi: 10.1128/mBio.02975-19 (PMC6946804; doi:10.1128/mBio.02975-19)
Supplement: FIG S5 [file mBio.02975-19-sf005.pdf]

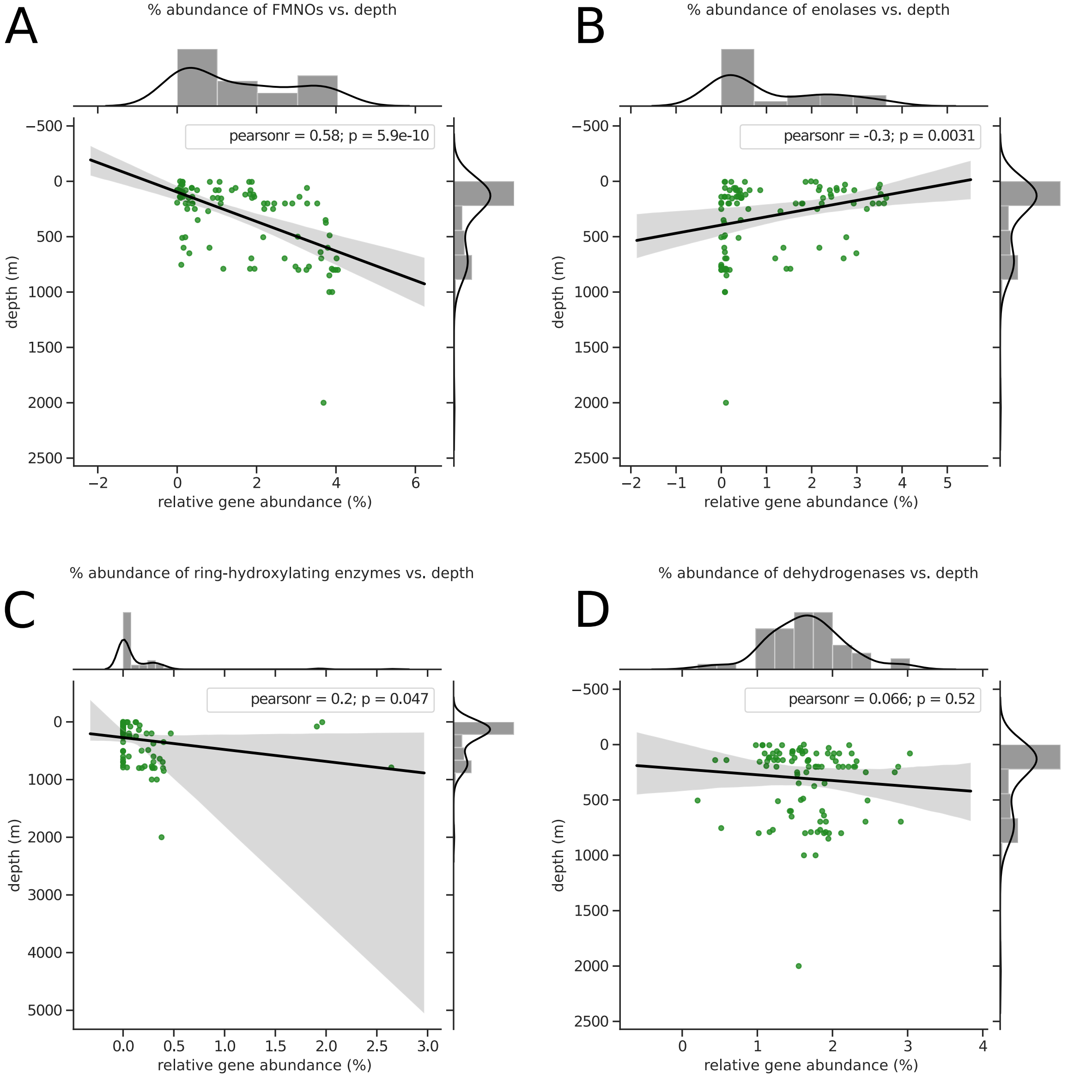

A
% abundance of FMNOs vs. depth
pearsonr = 0.58; p = 5.9e-10
depth (m)
relative gene abundance (%)
B
% abundance of enolases vs. depth
pearsonr = -0.3; p = 0.0031
depth (m)
relative gene abundance (%)
C
% abundance of ring-hydroxylating enzymes vs. depth
pearsonr = 0.2; p = 0.047
depth (m)
relative gene abundance (%)
D
% abundance of dehydrogenases vs. depth
pearsonr = 0.066; p = 0.52
depth (m)
relative gene abundance (%)
