# Supplementary material for: Pangenomics Analysis Reveals Diversification of Enzyme Families and Niche Specialization in Globally Abundant SAR202 Bacteria
Source: mBio. 2020 Jan 7;11(1):e02975-19. doi: 10.1128/mBio.02975-19 (PMC6946804; doi:10.1128/mBio.02975-19)
Supplement: FIG S7 [file mBio.02975-19-sf007.pdf]

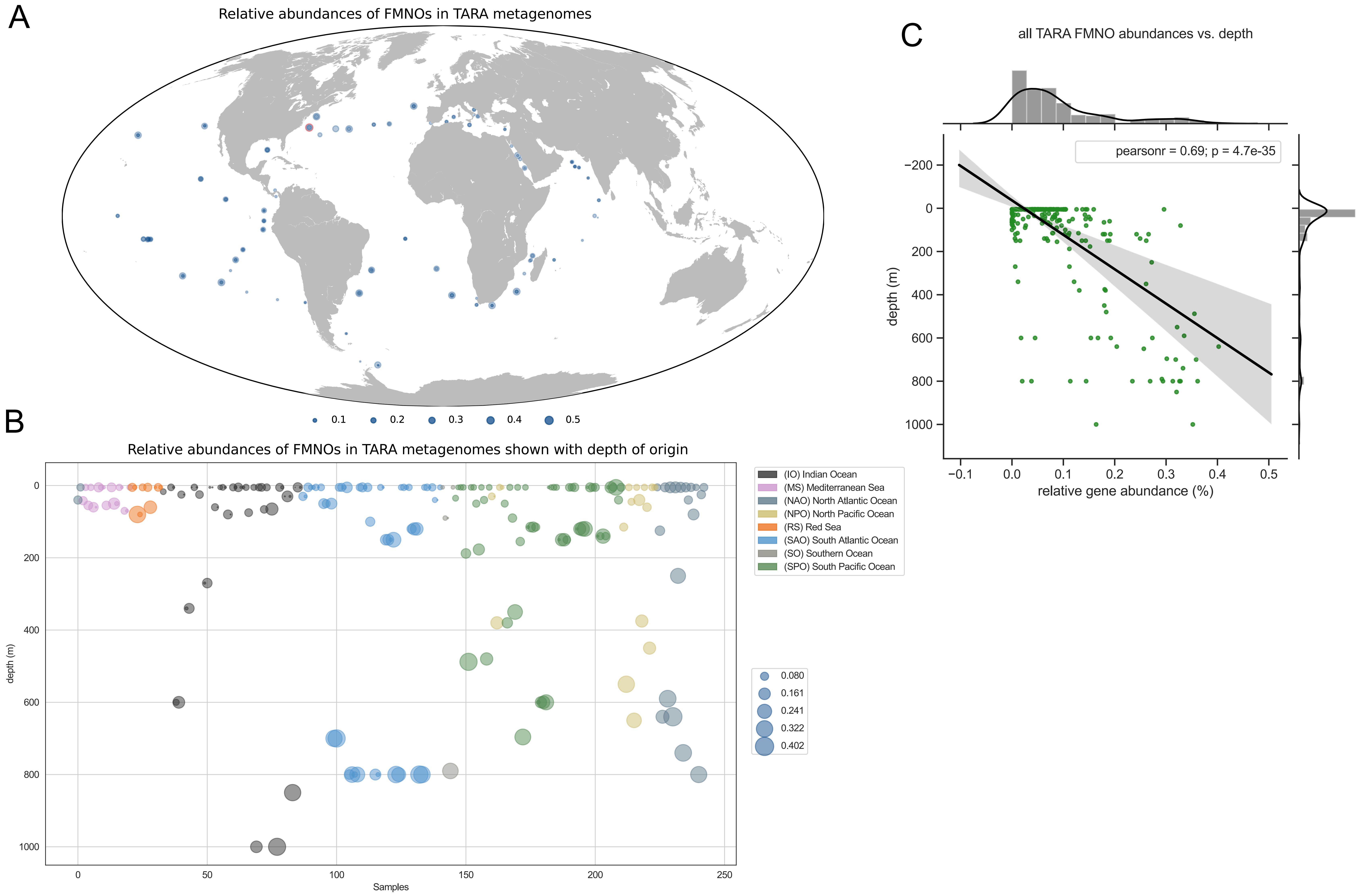
A
Relative abundances of FMNOs in TARA metagenomes
0.1
0.2
0.3
0.4
0.5
B
Relative abundances of FMNOs in TARA metagenomes shown with depth of origin
depth (m)
Samples
(IO) Indian Ocean
(MS) Mediterranean Sea
(NAO) North Atlantic Ocean
(NPO) North Pacific Ocean
(RS) Red Sea
(SAO) South Atlantic Ocean
(SO) Southern Ocean
(SPO) South Pacific Ocean
0.080
0.161
0.241
0.322
0.402
C
all TARA FMNO abundances vs. depth
pearsonr = 0.69; p = 4.7e-35
depth (m)
relative gene abundance (%)
