# Supplementary material for: Pangenomics Analysis Reveals Diversification of Enzyme Families and Niche Specialization in Globally Abundant SAR202 Bacteria
Source: mBio. 2020 Jan 7;11(1):e02975-19. doi: 10.1128/mBio.02975-19 (PMC6946804; doi:10.1128/mBio.02975-19)
Supplement: TEXT S1 [file mBio.02975-19-s0001.docx]

**Pangenomics reveal diversification of enzyme families and niche specialization in globally abundant SAR202 bacteria**

**Supplementary Online Materials and Methods**

## **Metagenomic sequencing of samples from deep-sea trenches**

## Each 6, 10 and 7 shotgun metagenomic sequencing libraries were constructed. The DNA extraction and shotgun metagenomic library constructions were described previously (1). Briefly, one third to half portion of the filter with microbial cells was minced and the microbial cells were lysed in FL Buffer (400 mM Tris-HCl [pH 8.0], 60 mM EDTA, 150 mM NaCl and 1% [wt/v] SDS), then, the resultant environmental DNA solution was further purified using a Power Soil DNA Isolation Kit (MoBio Lab) identical to the DNeasy PowerSoil Kit (QIAGEN, Hilden, Germany). Shotgun metagenomic libraries from the environmental DNA were constructed using a KAPA Hyper Prep Kit (for Illumina) (KAPA Biosystems, Wilmington, MA, USA) or Ovation SP+ Ultralow Library System (NuGEN, San Carlos, CA, USA). Metagenomic libraries were sequenced with an Illumina MiSeq Reagent Kit v3 (600 cycles).

## **Sample collection and metagenomic sequencing of samples from Bermuda Atlantic Time-series Study site**

## To collect the microbial biomass, about 4 L of seawater was filtered through 0.2 µm polyethersulfone membranes (Supor, Pall, East Hills, NY, USA). Membranes were stored at -80°C until further processing. Nucleic acids were extracted and purified as described earlier (2). During the extractions, nucleic acids were separated isopycnically by buoyant density centrifugation in cesium salts and the yields were measured by spectrophotometry. Metagenome library preparation and sequencing follows similar steps as described for the deep-sea trench metagenomes but the libraries were sequenced using an Illumina HiSeq instrument.

## **Phylogenomics analyses**

Hidden Markov Models (HMM) of a total of 5510 non-supervised orthologous groups belonging to the *Chloroflexi* phylum (ChloNOGs) download from the eggNOG database (dated May 4th, 2018) were searched against all 122 SAR202 genomes, 17 distant *Chloroflexi*, and 2 cyanobacteria genomes using hmmsearch with the E-value threshold set to 1. Resulting output files were searched with the “osu_extract_single_copy_genes.py” Python script (see link to Bitbucket repository) to extract all single-copy genes present in all genomes. The script only takes into consideration positive hmmsearch hits that are below E-value cutoff of 1e^-4^. This resulted in 639 chloNOGs matching the criterion. The number of single-copy marker genes were further reduced to include only the orthologous genes present in at least 60% of the 122 taxa. This reduced the number of marker genes to 49. Five of the chloNOGs among the 49 were found to be duplicates and only one copy of each were kept, further bringing the number of single-copy genes to 44. One of the SAGs (OSU_NS07) did not contain any of the 44 markers identified due to high incompleteness and was excluded from the phylogenomic analyses.

These 44 single-copy protein orthologs were individually aligned using mafft-linsi (3) and trimmed with BMGE (4) using the following parameters: -t AA -m BLOSUM30 -g 0.5 -b 3. IQ-Tree was used to infer single-gene phylogenies with the following parameters (-m LG+C10+F+G -bb 1000). Resulting maximum likelihood phylogenetic trees were first automatically analyzed using a Python script “osu_SAR202_single_gene_trees.py” (see link to Bitbucket repository), which automatically determines an outgroup to use, re-root the tree, determines monophyly of SAR202 members, and creates a PDF file output of these trees for manual inspection. Orthologous clusters in which all SAR202 members are monophyletic are included in the final concatenation. Gene trees in which SAR202 members are not monophyletic were then manually inspected to identify orthologous clusters that could be used in the final alignment concatenation. These gene trees are available on Figshare (DOI: 10.6084/m9.figshare.8478077).

Gene trees of orthologous clusters that display obvious patterns of horizontal gene transfer were excluded from further analyses. Members of *Dehalococcoidia* are closely related to SAR202 members and genes from *Dehalococcoidia* often cluster with those from SAR202 members. If SAR202 and *Dehalococcoidia* members form a monophyletic clade in an orthologous cluster, then it is included in the final list of orthologous clusters to be used in concatenations. Likewise, if a gene from a member of SAR202 falls outside of its otherwise monophyletic SAR202 clade, with low bootstrap supports (<95), then this orthologous cluster is also included in the final concatenation. This resulted in a total of 36 orthologous protein clusters included in the final concatenation.

Trimmed individual alignments were then concatenated to produce the final alignment, which resulted in a total of 5,466 characters. First, a Maximum Likelihood phylogenetic tree was inferred using IQ-Tree version 1.5.5 (5) using mixture models (-m LG+C60+F) and 1000 Ultrafast Bootstrap replicates (-bb 1000) (6). Next, a Bayesian phylogenetic tree was inferred using Phylobayes MPI version 1.7 (7) under the CAT-GTR+$\Gamma$ model, with four discrete $\Gamma$ categories. Four independent chains were run for more than 2700 generations, sampling every generation. Two of the independent chains reached convergence (burnin of 300 generations, maxdiff=0.28, effsize=126). Resulting Bayesian phylogenomic tree was visualized in Figtree (http://tree.bio.ed.ac.uk/software/figtree/), re-rooted with *Cyanobacteria*, and final aesthetics of the tree was improved using Inkscape. A full tree showing all leaves was also made using ETE3 Python library (8) (Supplemental Figure S10).

## **Phylogenetic analyses** **of FMNOs and enolases**

FMNO sequences in SAR202 genomes were identified by searching the SAR202 proteome against the conserved domain database using RPSBLAST with an E-value cutoff threshold of 1e^-4^. This identified 3135 FMNOs and the sequences found were clustered at 60% identity using USEARCH to reduce the number of sequences to be included in the tree (parameters: usearch -cluster_fast -id 0.6 -centroids). The clustering reduced the number of SAR202 FMNOs down to 506 sequences. To include the known diversity of FMNOs, 20,578 sequences belonging to Pfam accession PF00296 (Bac_luciferase) were downloaded from the Pfam website (pfam.xfam.org/PF00296). The sequences were filtered out to only include those within 200 to 500 residues long to exclude multi-domain proteins and separated into archaeal, bacterial, and eukaryotic sequences.

Due to the sheer number of proteins from the Pfam database, amino acid sequences were clustered at 60% using USEARCH to keep number of sequences tractable for the phylogenetic analyses. This resulted in 3,792 sequences from the Pfam database. They were further subsampled randomly using the seqtk tool to 1,858 sequences so that the total number of FMNOs to include in the tree amount to 2500. A total of 136 representative sequences of four subfamilies under the flavin-utilizing monooxygenases superfamily from conserved domain database (accession cd00347) were also downloaded (https://www.ncbi.nlm.nih.gov/Structure/cdd/cddsrv.cgi?uid=cd00347) to use these as references for determining subfamily boundaries in the final tree. The final set of sequences used to reconstruct the FMNO phylogeny includes 2500 sequences and they were aligned using mafft-linsi (3), trimmed with trimAl (9) with gap threshold set to 50%, and maximum likelihood phylogenetic tree built using IQ-Tree (version 1.5.5) (5) with the following parameters: -m LG+F+R9 -bb 1000.

To assess the diversity of enolases found in the SAR202 genomes, 6,562 sequences belonging to enolase superfamily was downloaded from the Pfam database (accession: PF00113). They were separated into bacterial, archaeal, and eukaryotic sequences, filtered to include sequences less than 500 residues long (to exclude multi-domain proteins), and individually clustered at 60% using USEARCH tool. A total of 97 reference sequences from four major subfamilies of enolases (enolase, MAL, MLE_like, and MR_like) were also downloaded from the CDD database (https://www.ncbi.nlm.nih.gov/Structure/cdd/cddsrv.cgi?uid=cd00308) to use them as references for determining subfamily boundaries in the final tree. A total of 2159 SAR202 proteins annotated as belonging to COG4948 (L-alanine-DL-glutamate epimerase and related enzymes of enolase superfamily) were clustered at 60% using USEARCH tool with same parameters used for clustering Pfam sequences. This reduced the numbers down to 265 representative clusters from the SAR202 genomes. Total number of sequences included in the enolase family phylogenetic tree is 913. Alignment, trimming, and phylogenetic inferences were done similarly as described for FMNOs. The -m parameter of IQ-Tree for enolases was LG+F+R10, a best-fitting model chosen by IQ-Tree model test (10).

### **Phylogenetic analyses of Bacteriorhodopsins**

HMM profile and non-supervised cluster of orthologous genes (NOGs) belonging to rhodopsins (COG5524) were downloaded from the eggNOG database and the profile was used to search against the SAR202 proteomes using HMMER with the E-value cutoff set to 1e^-3^. In addition, heliorhodopsin sequences from a recent study (11) were downloaded, aligned using mafft-linsi tool, trimmed with trimAl (3), and HMM profile was built using HMMER (12). HMM profile of heliorhodopsins was also used to search against the SAR202 proteomes. Resulting hits, along with reference rhodopsins from Pushkarev et al. (11) were aligned using mafft-linsi, trimmed with trimAl, and the resulting alignment was used to construct the maximum likelihood phylogenetic tree of rhodopsins using IQ-tree.

**Direct Cell Counts and Fluorescent *In Situ* Hybridization**

**Sample Collection**

Samples were collected via CTD rosette on cruise AE1703 in March 2017 and on AE1712 in July 2017. Samples taken on AE1703 were collected from 0, 40, 80, 120, 160, 200, 250, 300, 500, 600, 800m and 1000m and used to test the group specific probes for SAR202 designed in this study. Samples taken on AE1712 were collected from 0m, 40, 80, 120, 160, 200, 250, 300, 500, 600, 800, 1000, 1200, 1500, 2000, and 3000m. Seawater samples (40mL) were collected from the niskin and fixed with formalin (10%) for bacterioplankton abundance. Additional seawater samples (100mL) were collected in duplicate for fluorescent *in situ* hybridization analysis of the group specific probes for SAR202.

**Bacterioplankton abundance**

Seawater samples for bacterioplankton abundance were stored at -80°C until analysis. Seawater (10-20 mL) was filtered through a polycarbonate 0.2µm 25mm filter previously stained with with Irgalan black (SIGMA-Aldrich, St. Louis, MO USA) under light vacuum (5-7 mm Hg). The filters were then stained in darkness with 4’, 6-di-amidino-2-phenylindole dihydrochloride (5 ug ml^-1^ DAPI, SIGMA-Aldrich, St. Louis, MO USA, placed on a microscope slide using Resolve immersion oil and stored at -20C until enumeration (13). Total bacterioplankton abundance was determined using an AX70 epifluorescent microscope (Olympus, Tokyo, Japan) under ultraviolet excitation at 100x magnification. At least 400 cells (10 fields) were counted.

**Oligonucleotide probe development**

The group specific probes for SAR202 were designed for this study using ARB software (14) and the full-length SILVA database (15, 16). The oligonucleotide sequences were amended with cyanine 3 fluorescent dye (Supplemental Table 1).

**Fluorescent *in situ* hybridization (FISH)**

Samples for FISH were stored at -80°C until analysis. Samples were then thawed and 15-40mL of seawater were filtered onto 0.2 μm polycarbonate filters under gentle vacuum (100 mmHg) and stored at −20°C with desiccant.

The filters were cut into 6-8 pieces and washed in 95% ethanol and then analyzed by FISH according to previous protocols (17, 18). Reactions were performed in hybridization buffer (900 mM NaCl, 20 mM Tris [pH 7.4], 0.01% [wt/vol] sodium dodecyl sulfate [SDS], 15% formamide) as outlined in Supplemental Table 1. Seawater samples from AE1703 were used to determine dissociation curves for each of the three subgroups (Figure S9A). Optimal hybridization stringency was achieved by washing the membranes in hybridization wash (150 mM NaCl, 20 mM Tris [pH 7.4], 6 mM EDTA, 0.01% SDS) twice for 10 minutes. An experimentally determined temperature of dissociation (Td) specific for the group specific probes for the SAR202 Groups I, II and III was used for all group-specific SAR202 hybridization reactions (Supplemental Table 1). The filters were mounted with 20 μl of 1.67 μg ml−1 of DAPI in citifluor solution, sealed with nail polish and stored frozen in the dark until analysis (19).

Image analysis coupled with epifluorescent microscopy (Olympus AX70 microscope) was used to process FISH slides excited with Cy3 (550 nm) and UV wavelengths as previously described (20). The image capturing was performed using a Color Retiga Exi (QImaging®) digital camera (1392 x 1040 pixels) with Image Pro software (version 7.0; Media Cybernetics).

**Mass Spectrometry Analysis**

Seawater samples for mass spectrometry analysis were collected from the western Atlantic Ocean in 2013 at five stations (38º South, 45º West; 34.5º South, 42.5º West; 22.5º South, 33º West; 3º South, 28.5º West; 10º North, 55º West). Water samples were processed as previously described (21) and analyzed in negative ion mode on a 7T FT-ICR mass spectrometer (LTQ-FT Ultra, Thermo Fisher Scientific, Waltham MA) (22). Chemical transformations based on the gain or loss of oxygen were calculated using MetaNetter (21).

**Naming and Type strain designation of SAR202 Groups**

The phylogenomic tree supported earlier findings showing that SAR202 are a deeply-branching monophyletic group that radiates from within the *Chloroflexi*, possibly sister to *Dehalococcoides* (Fig. 1). Several deeply-branching subclades, Groups IV-VII, radiate near the base of the clade. Groups III, II and I appear in that order, ascending from the root. They are separated by large evolutionary distances and are the most abundantly represented SAR202 subgroups (Supplemental Table 1).

In their global analysis of bacterial phylogeny and systematics Parks et al. (2018) recommend that SAR202 be ranked as an order of the *Dehalococcoidia*. Accordingly, as we previously proposed that Group III be given the rank of class and assigned the name *Candidatus* “Monstramaria” (classis nov.), *Ca. “*Monstramariales” (ord. nov.) *Ca. “*Monstramariaceae” (fam. nov.; Landry et al., 2017), we suggest that the proposed order and family names be retained. Here we designate the genome of SAR202 isolate OSU_TB65 (NCBI Accession # VFHX00000000) as the type strain genome for the group III SAR202, to be given the name *Candidatus* “Monstramaris cthulu” (gen. nov, sp. nov.) The root of the class, order, family and genus name stems from the latin roots for 'Sea’ and ‘monster', to reflect the exclusive presence of members of the SAR202 clade to a number of marine and aquatic environments, and the species name cthulu (the greek root, cthonic, meaning subterranean) , is taken from the antagonist of the stories of H.P. Lovecraft that dwelled at the bottom of the ocean for an eternity, representing the age of the group as well as abyssal depths at which these cells reside, whose otherworldly appearance reflects the divergence of the group from other *Chloroflexi*, its cryptic genomic and metabolic features, and the inferred promiscuity of its enzyme systems. Material from the type strain was isolated from the 0.22-3 µm cell size fraction of seawater samples taken from 350 m depth (mesopelagic zone) in the North Pacific Ocean. The GC content of OSU_TB65 is 58.04%.

Given the separation of the subclades and the evolutionary distances between them in phylogenomic trees, below, we propose the following candidate names for the other SAR202 groups we report on here:

Group I will be designated *Candidatus* *“*Umibozuaceae” (fam. Nov.) after the mythical Japanese sea monster known as *Umibozu* from Japanese folklore, thought to haunt the shallow waters of fishing ports, reflecting the presence of this group in shallow waters. We designate genome of SAR202 isolate OSU_TB01 (NCBI accession # VFFO00000000) as *Ca “*Umibozu bosan*”* (Japanese, ‘Sea Monk’, ‘Monk’; gen. nov, sp. nov) and as the type strain genome for the group. Material from the type strain was isolated from the 0.22-3 µm cell size fraction of seawater samples taken from 115 m depth (Deep Chlorophyll Maximum) in the South Pacific Ocean. The GC content of OSU_TB01 is 44.99%.

We propose Group II be designated *Candidatus* “Scyllaceae”, fam. nov., after Scylla, from Greek mythology, a many headed monster living on one side of a narrow channel of water, its many-headed nature reflecting the proposed expansion enolase as well as FMNO paralogs to utilize DOM in the deep ocean. The source material for this group is SAR202 isolate OSU_TB21 (NCBI accession # VFGH00000000), and candidate name *Ca. “*Scylla pollaprosopa” , (‘polla’,’proposa’, Greek, ‘many’, ‘faces’; sp. nov. , gen. nov) Material from the type strain was isolated from the 0.22-1.6 µm cell size fraction of seawater samples taken from 600 m depth in the Indian Ocean. The GC content of OSU_TB21 is 61.25%.

Group IV *Candidatus* “Amphitriteaceae”, fam nov., named after Amphitrite, Greek goddess and queen of the sea. We designate the type strain of Group IV to be OSU_TB11 (NCBI accession # VFFX00000000). Material from the type strain was isolated from the 0.22-3 µm cell size fraction of seawater samples taken from 790 m depth in the Southern Ocean. The GC content of OSU_TB11 is 57.26%.

Group V, *Candidatus* “Cetusaceae”, fam nov., named for the ancient greek n kētŏs (κῆτος, plural kētē=kētea, κήτη, κήτεα), Latinized as cetus (pl. ceti or cetē = cetea), a great fish, a whale, shark, or sea monster. We suggest type material for this group as SAR202 isolate OSU_TB55 (NCBI accession # VFHN00000000) to be designated *Candidatus* “Cetusia infrequens” (gen. nov., sp. nov.; “infrequens”, rare) to reflect the sparsity of this group in the environment. Material for the type strain was isolated from the 0.22-3 µm cell size fraction of seawater samples taken from 140 m depth (mixed layer) in the South Pacific Ocean. The GC content of OSU_TB55 is 41.4%.

Group VI, *Candidatus* “Tiamataceae”, fam nov., named after Tiamat, the primordial goddess of the salt sea in Babylonian myth. Suggested name and type material is *Ca.* “Tiamatum marinus” and SAR202 isolate OSU_TB18 (NCBI accession # VFGE00000000), isolated from the 0.22-1.6 µm cell size fraction of seawater samples taken from 80 m depth in the Red Sea. The GC content of OSU_TB18 is 33.87%.

Group VII, *Candidatus* “Makaraceae” (fam nov.), named for a Hindu sea creature that is half terrestrial and half aquatic (see Colatriano et al., 2018, where the authors speculate that similar SAR202 found in coastal regions with expanded ring-hydroxylating dioxygenases may have acquired these genes from terrestrial sources). Suggested type material for this strain is SAR202 isolate OSU_TB62 (NCBI accession # VFHU00000000), to be given the candidate name *Ca. “*Makara kundala” (sp. nov. , gen. nov; species name derived from ring-shaped jewlery bearing her namesake). Material from the type strain was isolated from the 0.22-1.6 µm cell size fraction of seawater samples taken from 80 m depth in the Red Sea. The GC content of OSU_TB62 is 41.63%.

**References**

1. Hirai M, Nishi S, Tsuda M, Sunamura M, Takaki Y, Nunoura T. 2017. Library Construction from Subnanogram DNA for Pelagic Sea Water and Deep-Sea Sediments. Microbes Environ 32:336-343.

2. Giovannoni SJ, Rappe MS, Vergin KL, Adair NL. 1996. 16S rRNA genes reveal stratified open ocean bacterioplankton populations related to the Green Non-Sulfur bacteria. Proc Natl Acad Sci U S A 93:7979-7984.

3. Katoh K, Standley DM. 2013. MAFFT multiple sequence alignment software version 7: improvements in performance and usability. Mol Biol Evol 30:772-780.

4. Criscuolo A, Gribaldo S. 2010. BMGE (Block Mapping and Gathering with Entropy): a new software for selection of phylogenetic informative regions from multiple sequence alignments. BMC Evol Biol 10:210.

5. Nguyen LT, Schmidt HA, von Haeseler A, Minh BQ. 2015. IQ-TREE: a fast and effective stochastic algorithm for estimating maximum-likelihood phylogenies. Mol Biol Evol 32:268-274.

6. Hoang DT, Chernomor O, von Haeseler A, Minh BQ, Vinh LS. 2018. UFBoot2: Improving the Ultrafast Bootstrap Approximation. Mol Biol Evol 35:518-522.

7. Rodrigue N, Lartillot N. 2014. Site-heterogeneous mutation-selection models within the PhyloBayes-MPI package. Bioinformatics 30:1020-1021.

8. Huerta-Cepas J, Szklarczyk D, Forslund K, Cook H, Heller D, Walter MC, Rattei T, Mende DR, Sunagawa S, Kuhn M, Jensen LJ, von Mering C, Bork P. 2016. eggNOG 4.5: a hierarchical orthology framework with improved functional annotations for eukaryotic, prokaryotic and viral sequences. Nucleic Acids Res 44:D286-D293.

9. Capella-Gutierrez S, Silla-Martinez JM, Gabaldon T. 2009. trimAl: a tool for automated alignment trimming in large-scale phylogenetic analyses. Bioinformatics 25:1972-1973.

10. Kalyaanamoorthy S, Minh BQ, Wong TKF, von Haeseler A, Jermiin LS. 2017. ModelFinder: fast model selection for accurate phylogenetic estimates. Nat Methods 14:587-589.

11. Pushkarev A, Inoue K, Larom S, Flores-Uribe J, Singh M, Konno M, Tomida S, Ito S, Nakamura R, Tsunoda SP, Philosof A, Sharon I, Yutin N, Koonin EV, Kandori H, Beja O. 2018. A distinct abundant group of microbial rhodopsins discovered using functional metagenomics. Nature 558:595-599.

12. Eddy SR. 2011. Accelerated Profile HMM Searches. PLoS Comput Biol 7:e1002195.

13. Porter KG, Feig YS. 1980. The use of DAPI for identifying and counting aquatic microflora. Limnol Oceanogr 25:943-948.

14. Ludwig W, Strunk O, Westram R, Richter L, Meier H, Yadhukumar, Buchner A, Lai T, Steppi S, Jobb G, Förster W, Brettske I, Gerber S, Ginhart AW, Gross O, Grumann S, Hermann S, Jost R, König A, Liss T, Lüssmann R, May M, Nonhoff B, Reichel B, Strehlow R, Stamatakis A, Stuckmann N, Vilbig A, Lenke M, Ludwig T, Bode A, Schleifer KH. 2004. ARB: a software environment for sequence data. Nucleic Acids Res 32:1363-71.

15. Pruesse E, Quast C, Knittel K, Fuchs BM, Ludwig WG, Peplies J, Glockner FO. 2007. SILVA: a comprehensive online resource for quality checked and aligned ribosomal RNA sequence data compatible with ARB. Nucleic Acids Research 35:7188-7196.

16. Glöckner FO, Yilmaz P, Quast C, Gerken J, Beccati A, Ciuprina A, Bruns G, Yarza P, Peplies J, Westram R, Ludwig W. 2017. 25 years of serving the community with ribosomal RNA gene reference databases and tools. J Biotechnol 261:169-176.

17. Morris RM, Rappé MS, Urbach E, Connon SA, Giovannoni SJ. 2004. Prevalence of *Chloroflexi*-related SAR202 bacterioplankton cluster throughout the mesopelagic zone and deep ocean. Applied and Environmental Microbiology 70:2836-2842.

18. Morris R, Rappé M, Connon S, Vergin K, Siebold W, Carlson C, Giovannoni S. 2002. SAR11 clade dominates ocean surface bacterioplankton communities. Nature 420:806-810.

19. Parsons RJ, Breitbart M, Lomas MW, Carlson CA. 2012. Ocean time-series reveals recurring seasonal patterns of virioplankton dynamics in the northwestern Sargasso Sea. ISME J 6:273-84.

20. Carlson CA, Morris R, Parsons R, Giovannoni SJ, Vergin K. 2009. Seasonal dynamics of SAR11 populations in the euphotic and mesopelagic zones of the northwestern Sargasso Sea. ISME Journal 3:283-295.

21. Longnecker K, Kujawinski EB. 2016. Using network analysis to discern compositional patterns in ultrahigh-resolution mass spectrometry data of dissolved organic matter. Rapid Communications in Mass Spectrometry 30:2388-2394.

22. Kido Soule MC, Longnecker K, Giovannoni SJ, Kujawinski EB. 2010. Impact of instrument and experiment parameters on reproducibility of ultrahigh resolution ESI FT-ICR mass spectra of natural organic matter. Organic Geochemistry 41:725-733.
